# Supplementary material for: Reducing cell intrinsic immunity to mRNA vaccine alters adaptive immune responses in mice
Source: Mol Ther Nucleic Acids. 2023 Oct 5;34:102045. doi: 10.1016/j.omtn.2023.102045 (PMC10591005; doi:10.1016/j.omtn.2023.102045)
Supplement: Document S1. Figures S1–S3 [file mmc1.pdf]

OMTN, Volume *34*

Supplemental information

Reducing cell intrinsic immunity to mRNA vaccine alters adaptive immune responses in mice

Ziyin Wang, Egon J. Jacobus, David C. Stirling, Stefanie Krumm, Katie E. Flight, Robert F. Cunliffe, Jonathan Mottl, Charanjit Singh, Lucy G. Mosscrop, Leticia Aragão Santiago, Annette B. Vogel, Katalin Kariko, Ugur Sahin, Stephanie Erbar, and John S. Tregoning

Supplemental Materials

Table S1: Immunisation with D1-uRNA induces a range of genes associated with inflammation. BALB/c mice were immunised intramuscularly with 10 µg mRNA encoding HA from H1 influenza. The mRNA was either D1-uRNA, cC1-modRNA or D1-modRNA; responses were compared to buffer only. Gene list of significant DEG in lymph nodes 24 hours after immunisation.

Figure S1

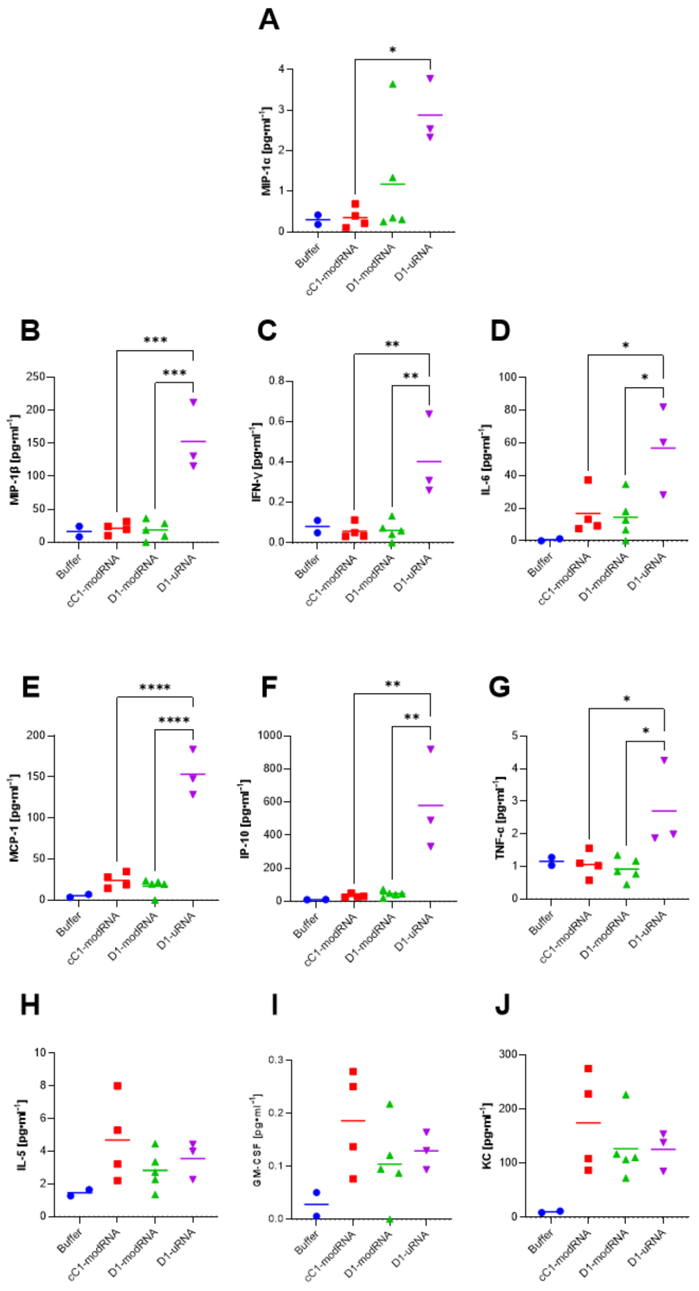

Figure S1. Immunisation with unmodified mRNA induces a significantly greater systemic inflammatory response 6 hours after immunisation. BALB/c mice were immunised intramuscularly with 10µg mRNA expressing HA from H1 influenza. The mRNA was either D1-uRNA, D1-modRNA, cC1-modRNA; responses were compared to buffer only. Blood was collected 6 hours after immunisation and measured for cytokines by MSD. Individual cytokines (A-J). * $p<0.05$, ** $p<0.01$, *** $p<0.001$, **** $p<0.0001$ as indicated by One Way ANOVA with post test.

Figure S2

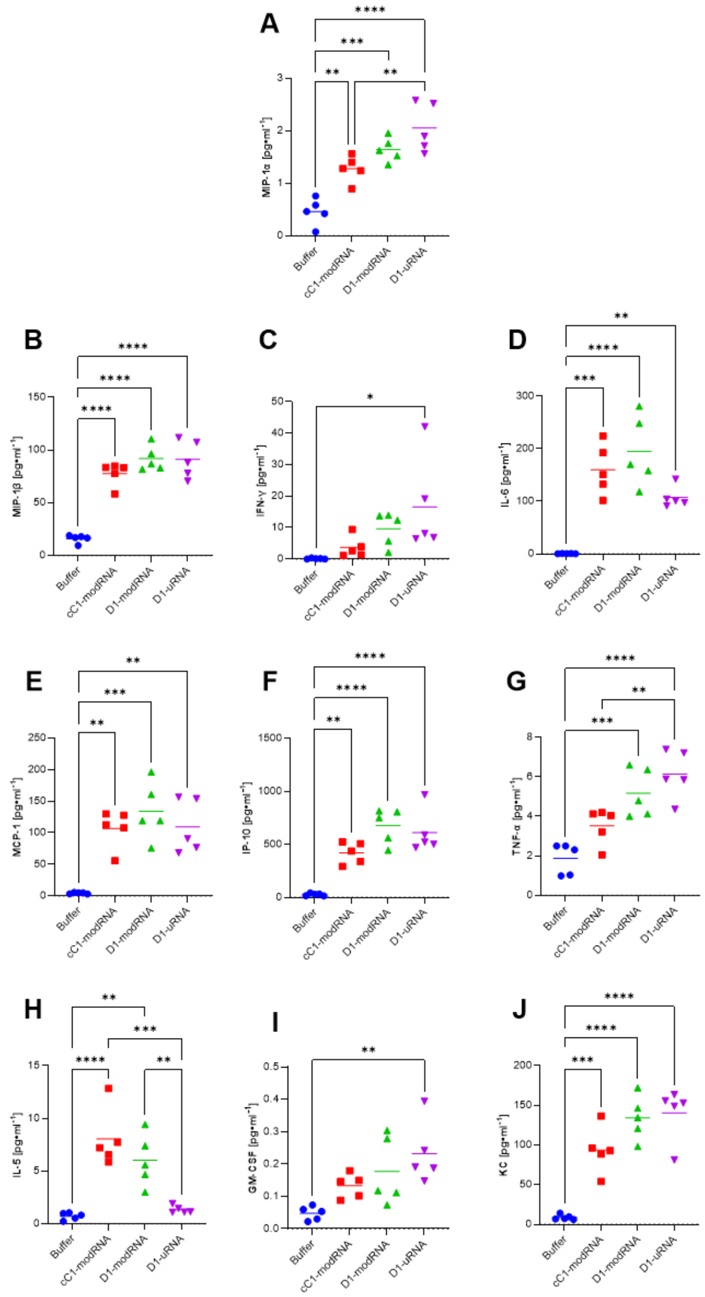

Figure S2. Immunisation with unmodified mRNA induces a significantly greater systemic inflammatory response 24 hours after immunisation. BALB/c mice were immunised intramuscularly with 10µg mRNA expressing HA from H1 influenza. The mRNA was either D1-uRNA, D1-modRNA, cC1-modRNA; responses were compared to buffer only. Blood was collected 24 hours after immunisation and measured for cytokines by MSD. Individual cytokines (A-J). * $p<0.05$, ** $p<0.01$, *** $p<0.001$, **** $p<0.0001$ as indicated by One Way ANOVA with post test.

Figure S3

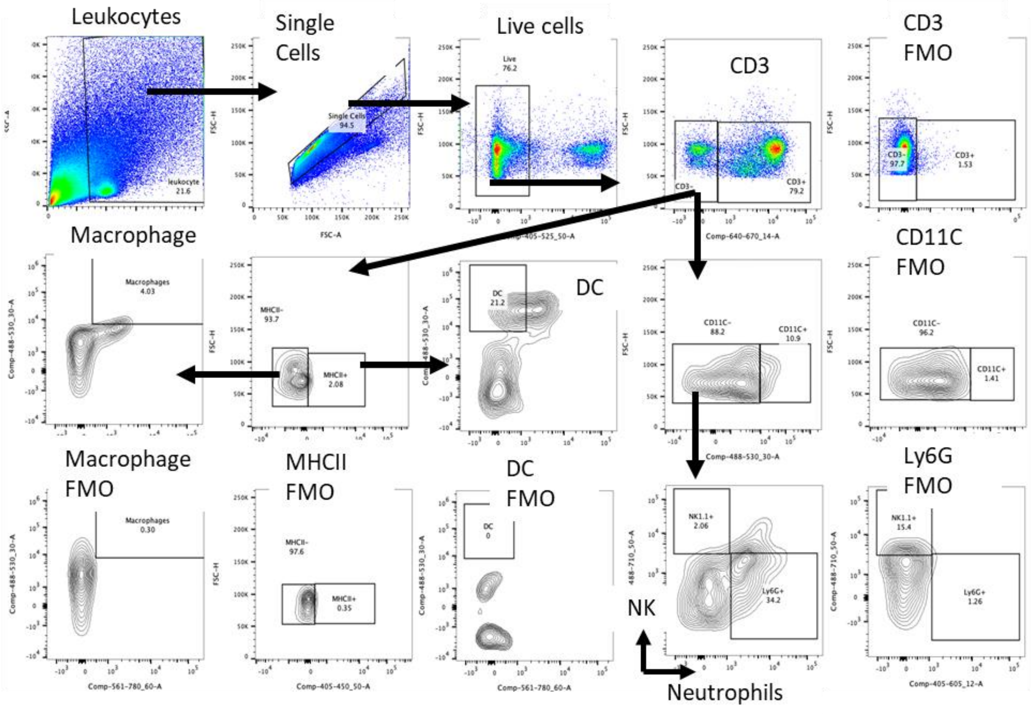

Figure S3. Gating strategy for flow cytometry analysis of leukocyte phenotypes in murine muscle and lymph nodes. Cells were isolated from lymph node and muscles after immunization and stained for flow cytometry. Analysis on FlowJo with sample gates demonstrated.
