# Supplementary material for: Reducing cell intrinsic immunity to mRNA vaccine alters adaptive immune responses in mice
Source: Mol Ther Nucleic Acids. 2023 Oct 5;34:102045. doi: 10.1016/j.omtn.2023.102045 (PMC10591005; doi:10.1016/j.omtn.2023.102045)
Supplement: Document S2. Article plus supplemental information [file mmc3.pdf]

Molecular Therapy
Nucleic Acids
Original Article

Reducing cell intrinsic immunity to mRNA vaccine alters adaptive immune responses in mice

Ziyin Wang,[1,3] Egon J. Jacobus,[2,3] David C. Stirling,[1] Stefanie Krumm,[2] Katie E. Flight,[1] Robert F. Cunliffe,[1] Jonathan Mottl,[2] Charanjit Singh,[1] Lucy G. Mosscrop,[1] Leticia Aragão Santiago,[2] Annette B. Vogel,[2] Katalin Kariko,[2] Ugur Sahin,[2] Stephanie Erbar,[2,4] and John S. Tregoning[1,4]

[1]Department of Infectious Disease, Imperial College London, London W2 1PG, UK; [2]BioNTech SE, An der Goldgrube 12, 55131 Mainz, Germany

The response to mRNA vaccines needs to be sufficient for immune cell activation and recruitment, but moderate enough to ensure efficacious antigen expression. The choice of the cap structure and use of N1-methylpseudouridine (m1Ψ) instead of uridine, which have been shown to reduce RNA sensing by the cellular innate immune system, has led to improved efficacy of mRNA vaccine platforms. Understanding how RNA modifications influence the cell intrinsic immune response may help in the development of more effective mRNA vaccines. In the current study, we compared mRNA vaccines in mice against influenza virus using three different mRNA formats: uridine-containing mRNA (D1-uRNA), m1Ψ-modified mRNA (D1-modRNA), and D1-modRNA with a cap1 structure (cC1-modRNA). D1-uRNA vaccine induced a significantly different gene expression profile to the modified mRNA vaccines, with an up-regulation of *Stat1* and *RnaseL*, and increased systemic inflammation. This result correlated with significantly reduced antigen-specific antibody responses and reduced protection against influenza virus infection compared with D1-modRNA and cC1-modRNA. Incorporation of m1Ψ alone without cap1 improved antibodies, but both modifications were required for the optimum response. Therefore, the incorporation of m1Ψ and cap1 alters protective immunity from mRNA vaccines by altering the innate immune response to the vaccine material.

INTRODUCTION

The coronavirus disease 2019 (COVID-19) pandemic has led to a flurry of vaccine developments and roll outs, including a number of vaccine platforms that had not previously been licensed.[1,2] One of the platforms that went from a promising pre-clinical approach to a highly effective product was mRNA-based vaccines. There are now two mRNA vaccines widely in use: BNT162b2 (Comirnaty, manufactured by Pfizer-BioNTech) and mRNA-1273 (Spikevax, manufactured by Moderna).[3,4] A key underpinning technology that contributed to the success of these vaccines was the replacement of uridine-containing mRNA (uRNA) with m1Ψ (modRNA).[5] As well as the COVID-19 vaccines,[6] modRNA has been used in a wide number of pre-clinical studies against viruses, such as influenza[7] and Zika,[8] and other applications, such as cancer[9] and experimental autoimmune encephalomyelitis.[10] It is also being tested in a number of clinical trials for other infections, such as influenza (NCT05052697) and respiratory syncytial virus (NCT05127434). Understanding more about the impact of m1Ψ modification on the innate immune response to mRNA vaccines would help in the development of more effective vaccines.

The recognition of foreign RNA by the host immune response can reduce mRNA translation. Foreign RNA is sensed by a range of innate immune receptors, such as Toll-like receptors (TLR3 and TLR7), OAS-1, and retinoic acid-inducible gene-I (RIG-I)-like receptors, such as melanoma differentiation-associated protein 5 and RIG-I.[11] For mRNA vaccines, evading the recognition of the foreign RNA may result in better translation efficiency in the injected tissue and increase the desired downstream adaptive immune response. The replacement of uridine with m1Ψ in mRNA suppressed recognition of the delivered RNA by TLRs, with an associated reduction in the production of inflammatory cytokines,[5] and led to higher protein translation than unmodified mRNA.[12] Another structural element important for mRNA stability and translation efficiency is the 5′ cap. Different 5′ caps for co-translational capping of *in vitro* transcribed mRNA production are available, among which the cap0 ß-S-ARCA cap (D1) has been shown to enhance RNA stability and translational efficiency,[13] while the cap1 CleanCap (cC1) offers reduced immune sensing.[14] An important additional step is the removal of double-stranded RNA (dsRNA) molecules, which are a low-level by-product during RNA production, from the vaccine preparation,[15] as dsRNAs are potent inducers of innate immunity.[16]

https://doi.org/10.1016/j.omtn.2023.102045.

[3]These authors contributed equally

[4]These authors contributed equally

Correspondence: Egon J. Jacobus, PhD, BioNTech SE, An der Goldgrube 12, 55131 Mainz, Germany.
E-mail: egon.jacobus@biontech.de
Correspondence: John S. Tregoning, PhD, Department of Infectious Disease, Imperial College London, London W2 1PG, UK.
E-mail: john.tregoning@imperial.ac.uk

This is an open access article under the CC BY license (http://creativecommons.org/licenses/by/4.0/).

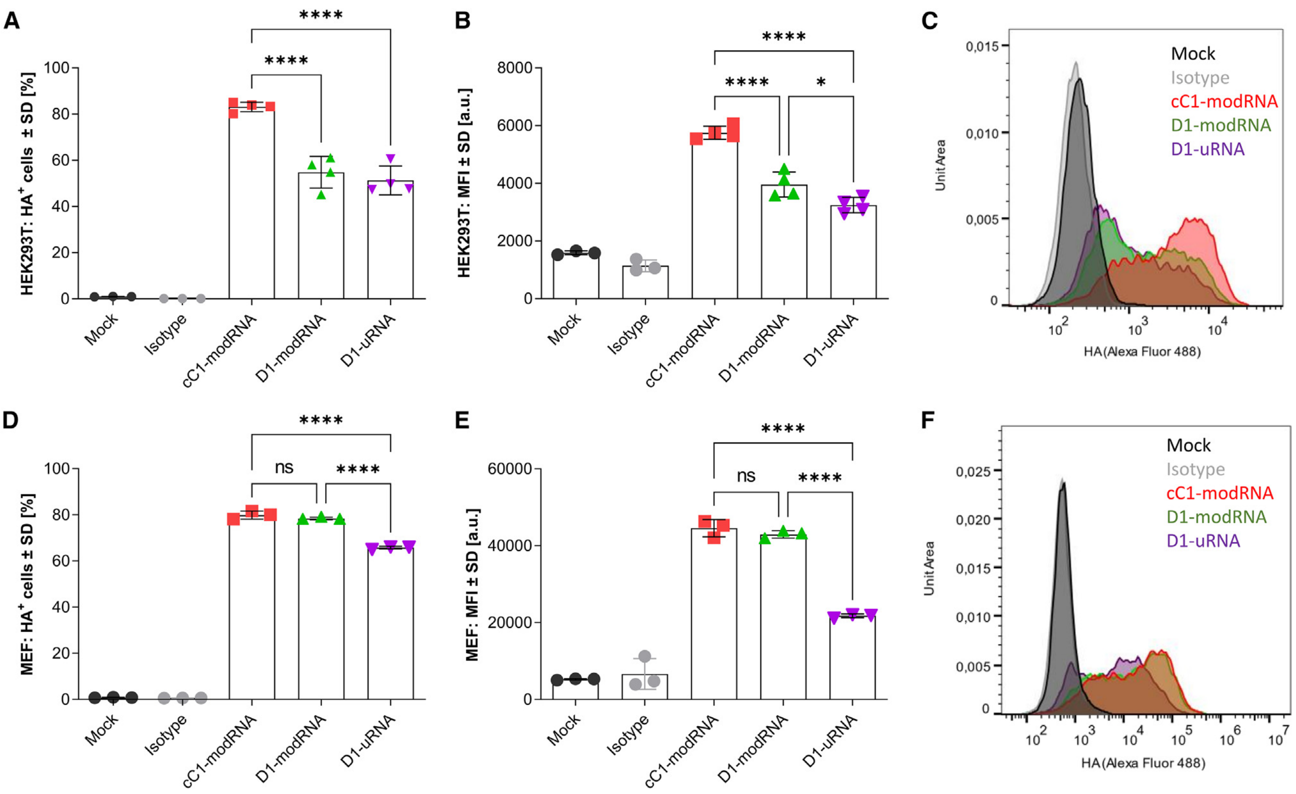

Figure 1. Modifications of the mRNA format influence expression in vitro

(A–D) HEK293T (A, B) and MEF (C, D) cells were either transfected with different mRNA formats encoding H1 influenza HA, or mock transfected. Eighteen hours after transfection, cells were stained with ant-HA primary antibody or isotype control (IgG2a kappa), and analyzed by flow cytometry. Expression was assessed by displaying the frequency of HA-positive cells (A, D) and their mean fluorescence intensity (MFI, B, E) as mean and standard deviation and overlay plots represent one representative sample (C and F). One-way ANOVA with Tukey's multiple comparison was performed between transfected groups, n = 4 for HEK293T cells and n = 3 for MEFs. *$p < 0.05$, ****$p < 0.0001$. ns, not significant.

Recognition of foreign RNA activates the type I interferon (IFN) system,[17] which induces an anti-viral state. The IFN-induced anti-viral state inhibits protein translation through the up-regulation of IFN stimulated genes.[18] In a recent systems immunology study, up-regulation of type I IFN-associated genes has been observed in volunteers immunized with the mRNA vaccine BNT162b2.[19]

To dissect the innate immune response that suppresses translation of injected RNA or dampens the antigen-specific immune response, we performed a comparison of mRNA vaccine formats against an influenza virus antigen. We compared *in vitro* transcribed β-S ARCA capped (D1) mRNA with m1Ψ (m1Ψ-modified mRNA [D1-modRNA]) and without nucleoside modification (D1-uRNA). As a third RNA format, we combined two approaches to further reduce immune sensing by producing an m1Ψ mRNA which incorporated cC1 and was produced with an *in vitro* transcription protocol that yields reduced dsRNA impurities (D1-modRNA with a cap1 structure [cC1-modRNA]). Our hypothesis was that sensing of the different mRNA vaccines by the innate immune system would have an impact on the downstream adaptive immune response. We saw that the D1-uRNA RNA format led to a significantly different gene expression profile in the lymph node than the D1-modRNA and cC1-modRNA formats. This was associated with increased inflammation and a significantly reduced adaptive immune response to the encoded antigen. In mice lacking IFN-α/β receptor (IFNAR), there was an altered response to the vaccine, with increased T cell responses. Among the mRNA formats tested, cC1-modRNA vaccine generated the highest anti-hemagglutinin (HA) antibody titers and controlled best an influenza viral challenge in mice.

RESULTS

Incorporation of m1Ψ into mRNA significantly increases *in vitro* antigen expression

Previous studies have shown that the incorporation of m1Ψ into mRNA increases protein translation.[12] We wanted to investigate whether this was the case with mRNA encoding influenza virus HA, and whether the additional incorporation of Cap1 and dsRNA-removal step have an impact, as these are hypothesized to reduce sensing by pattern recognition receptors. Initially, we investigated the protein expression in the IFN-deficient cell line HEK293T. Cells were transfected with 80 ng mRNA and expression of HA was measured by flow cytometry 18 h later. There were significantly more HA-positive cells after transfection with cC1-modRNA (Figure 1A), and positive

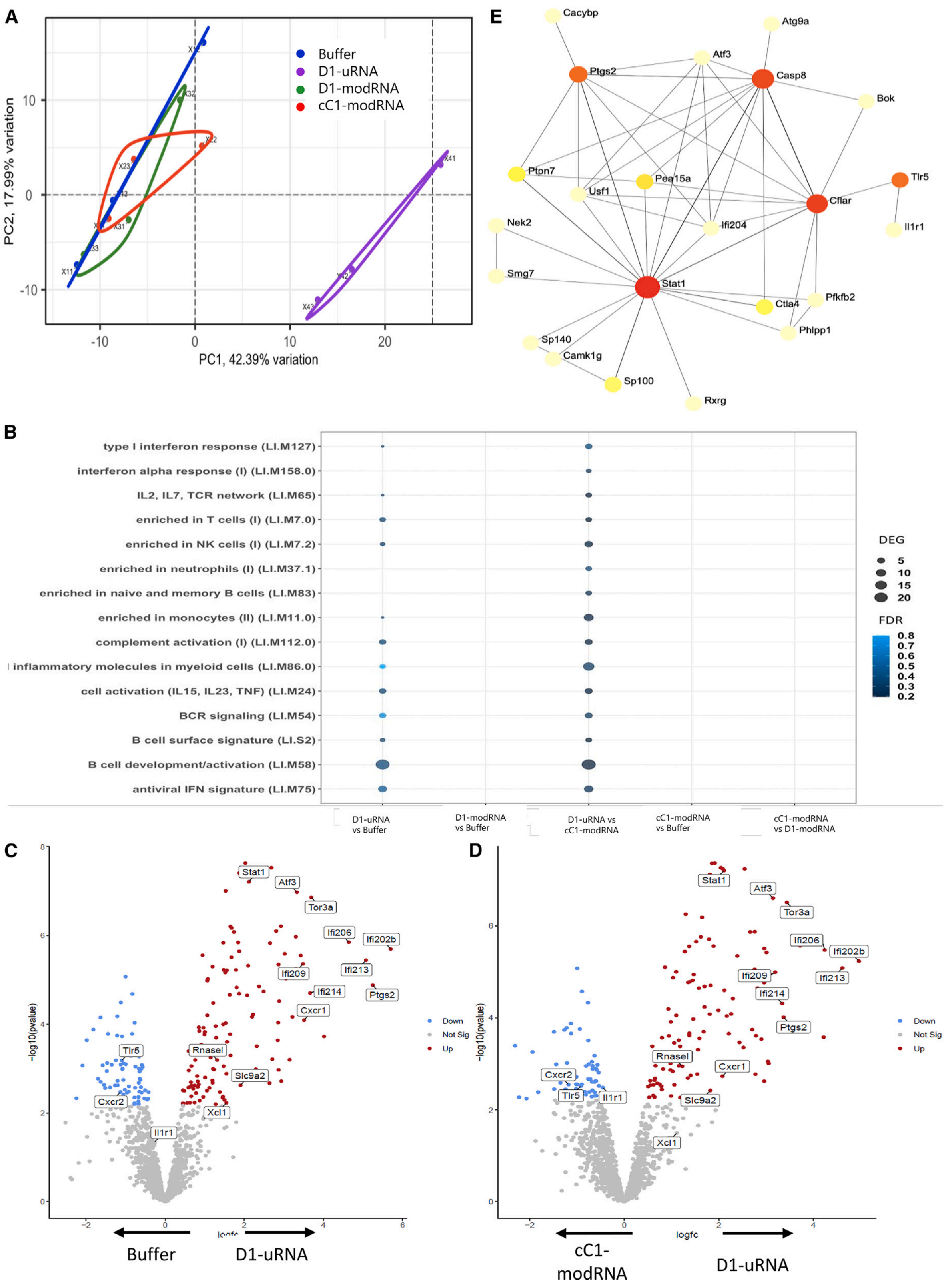

(legend on next page)

cells expressed a higher level of HA than D1-modRNA and D1-uRNA transfected cells (measured by MFI, Figures 1B and 1C). We also transfected mouse embryonic fibroblasts (MEFs) that, unlike HEK cells, are IFN sensitive. MEFs transfected with 150 ng of the modified RNAs, either D1-modRNA or cC1-modRNA, had a significantly greater number of HA-positive cells (Figure 1D), and those that were positive had higher levels than D1-uRNA transfected cells (Figures 1E and 1F). These *in vitro* data indicate that the use of m1Ψ in the mRNA had largest impact on antigen expression in an IFN-sensitive system, whereas the incorporation of Cap1 and dsRNA removal by cellulose purification (cC1-modRNA) only increased antigen expression significantly in an IFN-deficient system. As seen in other studies, modified mRNA showed better translation *in vitro*.[20]

Incorporation of m1Ψ into mRNA significantly alters the inflammatory response to mRNA vaccines

Having observed an effect on *in vitro* expression, we investigated the impact of incorporating m1Ψ on the immune response to an influenza mRNA vaccine *in vivo*. One hypothesis was that the D1-uRNA would trigger a stronger type I IFN response, which inhibits expression of the vaccine-encoded antigen. To test this, we looked at the impact of mRNA vaccine format and clean-up on the RNA transcriptome in the draining lymph node 6 h after a single immunization of 10 μg of each mRNA platform intra-muscularly (i.m.). In a global analysis of the response by principal component analysis (PCA), there was a clear separation of the D1-uRNA group from the other groups (Figure 2A), indicating a difference between the responses. Most of the variance was driven by principal component 1 (50%) and this was explained mostly by genes associated with the type I IFN response.

We investigated the blood transcription modules[21] into which the differentially expressed genes (DEGs) were grouped (Figure 2B). There were significant changes between D1-uRNA and the buffer group in several blood transcription modules as defined by Li et al.[22]; this included B cell development (LI.M58) and antiviral IFN signature (LI.M75). Similar differences were seen when cC1-modRNA was compared with D1-uRNA, with differences in the type I IFN response module (LI.M127), inflammatory molecules in myeloid cells (LI.M86.0) and antiviral IFN signature (LI.M75). No differences were seen between either cC1-modRNA or D1-modRNA and buffer.

We then compared individual genes that were significantly different, first between the D1-uRNA and a buffer-only group (Figure 2C). There were 80 DEGs that were up-regulated (Table S1); these included the type I IFN signaling molecule *Stat1*, multiple members of the p200 family (*Ifi202b*, *Ifi211*, *Ifi206*, *Ifi203*, *Ifi209*, *Ifi205*, *Ifi208*, *Ifi214*, *Ifi204*, *Ifi207*, *Ifi213*, and *Mndal*), the chemokine *Xcl1*, the chemokine receptors *Ackr1* and *Cxcr1*, and the immune checkpoint regulator *Ctla4* and *RnaseL*. There were 57 genes that were significantly greater in the PBS control. A very similar pattern was seen when comparing D1-uRNA with cC1-modRNA, there were 71 DEGs that were significantly up-regulated in D1-uRNA compared with cC1-modRNA, with many of the same genes as seen in the comparison of D1-uRNA and buffer (Figure 2D). There were 53 genes that were significantly greater in the cC1-modRNA group. When buffer and cC1-modRNA were compared, there were no significant DEG. Mapping the interactions of the DEG between D1-uRNA and buffer indicated that Stat1 was as an important active hotspot, suggesting its role as a master regulator (or the importance of IFN-signaling pathways) during the responses induced by the vaccine (Figure 2E).

We explored whether the changes in the transcriptome after immunization were associated with an impact on systemic inflammation. Immunization led to a mild but transient weight loss 1 day after immunization, with the D1-uRNA group losing more weight (Figure 3A), indicating potential systemic effects. To investigate mediators associated with inflammation, we measured the cytokine response in blood 6 and 24 h after immunization of mice receiving a 10-μg dose i.m. of either mRNA platform. The cytokine response to D1-uRNA was elevated with the induction of a significantly greater level of different analytes compared with the other mRNA vaccines tested (Figures 3B and S1), including MIP-1A, MIP-1B, IFN-γ, interleukin (IL)-6, MCP-1, IP-10, and tumor necrosis factor (TNF). There was no significant difference in IL-5, granulocyte macrophage colony stimulating factor (GM-CSF), or KC (Figures S1H–S1J). Blood samples were also collected 24 h after immunization (Figures 3C and S2). At this time point, all vaccinated groups had significantly elevated levels of cytokines compared with the buffer group, with significantly increased levels of MIP-1A, MIP-1B, IL-6, MCP-1, IP-10, and KC (Figures S1A, S1B, S1D–S1F, and S1J). Only D1-uRNA had a significantly higher level of IFN-γ and GM-CSF than buffer (Figures S2C and S2I). The D1-uRNA group had significantly higher levels of MIP-1A (Figure S2A) and TNF (Figure S2G) than the cC1-modRNA group. IL-5 was significantly greater in the cC1-modRNA and D1-modRNA groups than either buffer or D1-uRNA (Figure S2H). These data indicate that all mRNA vaccines tested induce a systemic inflammatory response, but the kinetics and magnitude of the response to the D1-uRNA are faster than in the other mRNA vaccines.

We then investigated the impact on cellular infiltration into the injection site (the muscle) and the local draining lymph nodes. Tissues were collected 24 h after immunization. There was a visual difference in the muscle and lymph nodes, with some evidence of bloody

Figure 2. Immunization with unmodified mRNA induces a significantly different transcriptomic response in the lymph node to m1Ψ containing mRNA
(A–D) BALB/c mice were immunised intramuscularly with 10 μg mRNA encoding HA from H1 influenza. The mRNA was either D1-uRNA, cC1-modRNA or D1-modRNA; responses were compared to buffer only. Lymph nodes were collected at 6 hours after immunisation and processed for RNA-Seq. Principal component analysis (PCA) of RNA changes (A). Grouping of responses as blood transcription modules (BTM, B). Individual significant differentially expressed genes (DEG), shown between buffer and D1-uRNA (C) and cc1-modRNA and D1-uRNA (D). Gene-gene interactions for DEG in D1-uRNA group (E). N=3 mice per group. FDR, false discovery rate.

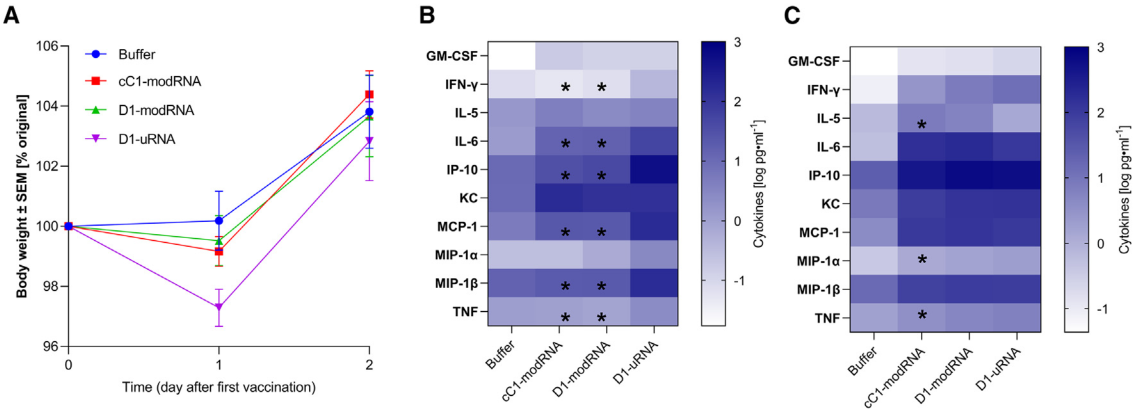

Figure 3. Immunization with unmodified mRNA induces a significantly greater systemic inflammatory response 6 h after immunization
(A–C) BALB/c mice were immunized intramuscularly with 10 μg mRNA encoding HA from H1 influenza. The mRNA was either D1-uRNA, cC1-modRNA or D1-modRNA. Weight change after immunization (A). Blood was collected at 6 h (B) and 24 h (C) after immunization and measured for cytokines by MSD. N = 5 mice per group. *on heatmap $p < 0.05$ compared with D1-uRNA group by one-way ANOVA with Tukey's post hoc test.

infiltrate into the muscles after cC1-modRNA and relatively enlarged lymph nodes in the D1-uRNA group. The lymph node and muscle tissues were digested, and live cells were counted. There were increased cell numbers in the muscle (Figure 4A) of the group immunized with cC1-modRNA and increased cell numbers in the lymph node of the D1-uRNA group (Figure 4B), suggesting a different kinetics of cell recruitment. There were slight differences in the cell types detected. While there was no difference MHCII^{+}CD11C^{+} cell counts (dendritic cells) in the muscle (Figure 4C), the D1-modRNA group had significantly more dendritic cells in the lymph node (Figure 4D). There was no difference in macrophages in muscle or lymph node (Figures 4E and 4F). Interestingly, there were more neutrophils in the muscle after immunization with any of the mRNA formats than in the buffer control (Figure 4G), and there was a trend toward more neutrophils in the lymph node of mice immunized with D1-uRNA RNA (Figure 4H). There were no differences in T cell recruitment to in the muscle (Figure 4I), but there was a trend toward more T cells in the lymph nodes of the D1-uRNA-immunized mice than the other groups (Figure 4J).

Systemic inflammation after mRNA vaccination is associated with a dampened adaptive immune response

Having seen differences in the inflammatory response to the different mRNA vaccines, we wanted to investigate whether this was associated with differences in adaptive immune responses. Mice were immunized in a prime-boost regime at days 0 and 21 with 10 μg mRNA, comparing the three mRNA vaccine types. Blood was collected 24 h after first immunization and cytokines measured by multiplex assay. As seen previously (Figure 3), there was a greater systemic inflammatory response in mice immunized with D1-uRNA than cC1-modRNA, with significantly elevated levels of IL6, KC, and TNF, but significantly less IL-5 (Figure 5A). Anti-H1 HA influenza antibodies were significantly greater in both the cC1-modRNA and the D1-modRNA groups than the D1-uRNA group after both prime (Figure 5B) and boost (Figure 5C) immunization. Hemagglutination inhibition assay (HAI) titers reflected ELISA titers (Figure 5D). We compared the cytokine response at 24 h after the first immunization and the antibody response after the prime or boost immunizations (Figure 5E). There was a strong negative correlation between levels of KC (Figure 5F) and TNF (Figure 5G), and antibody response 21 days after prime immunization; this relationship continued to the antibody response after a boost immunization. Interestingly, the only cytokine with a strong positive correlation with antibody was IL-5 (Figure 5H). Influenza HA-specific T cell responses measured at day 56 by ELISpot. and there were no differences between groups (Figure 5I). There was a correlation between most cytokines and T cell responses, with a significant but weak correlation between IP-10 and HA specific T cells (Figure 5J) when comparing the immunized groups, although this is anchored in the naive group.

To assess the role of IFN sensing on the performance of RNA vaccines, we compared responses to cC1-modRNA and D1-uRNA in C57BL/6 mice with and without αIFNAR treatment, or *Ifnar*$^{-/-}$ gene knockout mice. Mice were immunized intramuscularly with 1 μg vaccine in a prime boost regime with 28 days between injections; before each immunization, antibody-treated mice were given 1 mg MAR1 intraperitoneally. Blood was collected 6 h after immunization for analysis of cytokines. Blocking signaling through IFNAR—either in knockout mice or by antibody treatment—had no impact on the cytokine response to the cC1-modRNA (Figure 6A). However, there was a significant reduction in IP-10 (Figure 6B) and TNF (Figure 6C) in the IFNAR-blocked D1-uRNA groups. Interestingly, there was also an increase in IL-5 (Figure 6D) and IL-6 (Figure 6E) in *Ifnar*$^{-/-}$ mice compared with untreated mice. Other cytokines measured showed no significant change (Figure 6A). Sera were collected at day 42 and anti-HA responses measured by ELISA. The antibody responses were low across all animals, indistinguishable from the control group, which may reflect different responses in different mouse backgrounds (C57BL/6 in these studies to match the IFNAR$^{-/-}$) and the lower amount of RNA used compared with other studies (Figure 6F). However, there

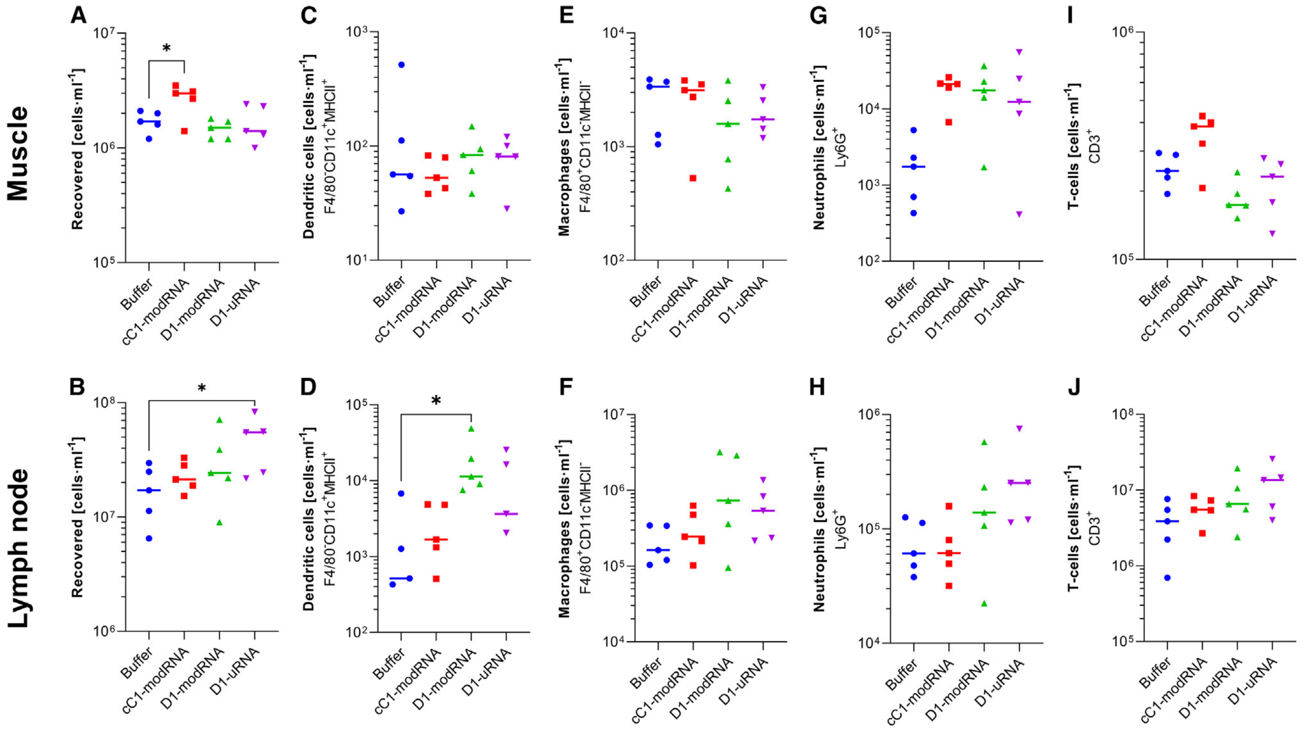

Figure 4. Immunization with unmodified mRNA leads to different cell recruitment into the muscle and lymph node

(A–J) BALB/c mice were immunized intramuscularly with 10 μg mRNA encoding HA from H1 influenza; the mRNA was either D1-uRNA, cC1-modRNA, or D1-modRNA. Muscles and lymph nodes were harvested at 24 h after immunization and processed for flow cytometry. Total live leukocyte cell count in recovered tissue (A and B), dendritic cells (C and D), macrophages (E and F), neutrophils (G and H), and T cells (I and J) assessed by flow cytometry and presented as a percentage of the live leukocytes in the tissue sample. The mean is indicated by horizontal line, N = 5 mice per group, each dot represents one animal, $^{*}p < 0.05$ by one-way ANOVA and Dunnet's post hoc test.

was a significant decrease in HA-specific T cell responses in the D1-uRNA-immunised IFNAR blocked mice (Figure 6G) and T cell responses correlated with IP-10 (Figure 6H).

Finally, we assessed the impact of different mRNA vaccine types on protection against influenza virus challenge. We tested three doses of the three different mRNA platforms: 10, 1, and 0.2 μg. As before, mice were immunized in a prime-boost regime, and blood collected at day 21 (after one dose) and day 42 (21 days after second dose). One dose of 10 μg cC1-modRNA or D1-modRNA led to significantly greater antibody levels than the D1-uRNA, although there was detectable antibody to all vaccines (Figure 7A). A similar effect was seen with 1 μg C1-modRNA, which was significantly greater than the D1-uRNA and did not induce any antibody response after prime (Figure 7B). There was no antibody response in any group after one dose of 0.2 μg mRNA (Figure 7C). After two doses of mRNA, all animals seroconverted, except for the 0.2-μg D1-uRNA group (Figures 7D–7F), with the 10-μg and 1-μg doses of cC1-modRNA group significantly greater than the D1-uRNA. There was also a significant difference between the cC1-modRNA and D1-modRNA in the 10-μg and 1-μg dosed groups observed (Figures 7D and 7E). At 21 days after the second dose, animals were infected intranasally with H1N1 influenza. The animals were assessed for signs of disease by assessing weight loss. All the 10-μg groups were protected against weight loss, and the experiment was terminated at day 4 after infection (Figure 7G). In the 1-μg study, all groups lost some weight after infection, but compared with the buffer control, the mice lost significantly less weight and the buffer group had to be culled at day 5 because they had reached the humane endpoint. The cC1-modRNA group lost significantly less weight than the D1-uRNA group (Figure 7H). In the 0.2-μg study, the cC1-modRNA group also lost significantly less weight than the D1-uRNA group, and two mice in the D1-uRNA group were culled on day 5 because of excess weight loss (Figure 7I). We also estimated viral load by measuring influenza M gene RNA levels in frozen lung tissue. In the 10-μg study, all immunized animals had significantly less M gene RNA in their lungs on day 4 after infection than the buffer control (Figure 7J). There was detectable M gene RNA in the lungs of the 1-μg and 0.2-μg immunized groups at the later time point of day 7 (there was no buffer group control at this time point because they were culled on day 5). There was no significant difference between the groups.

DISCUSSION

In the current study, we investigated the impact of the incorporation of m1Ψ, as well as the combination of m1Ψ, Cap1, and dsRNA purification into a D1-uRNA vaccine against influenza virus on antigen

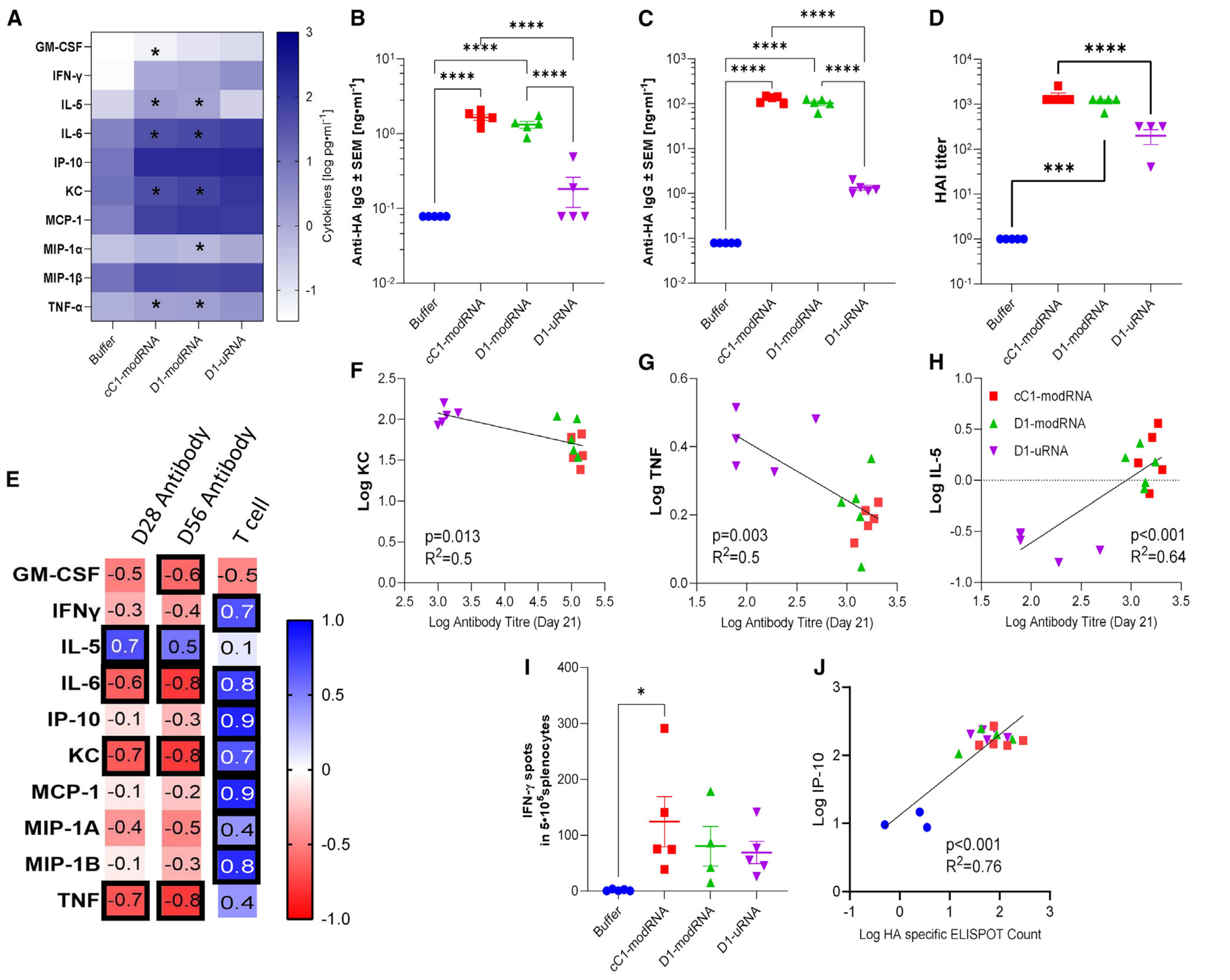

Figure 5. Inflammation after immunization with unmodified mRNA is associated with dampened adaptive immune responses

(A-J) BALB/c mice were immunized at day 0 and day 21 intramuscularly with 1 μg mRNA encoding HA from H1 influenza; the mRNA was either D1-uRNA, cC1-modRNA, or D1-modRNA. Heatmap of mean cytokine responses in blood 24 h after immunization (A). Blood was collected for analysis of HA specific antibody 21 days (B) and 42 days (C) after study start; 42 days after the study, spleens were collected and assessed for HA specific T cells by ELISPOT (D). Heatmap of correlation between antibody, T cells, and cytokines after prime, values are Pearson r (E), black boxes indicate significance $p < 0.05$. Correlation antibody with KC (F), TNF (G), IFN-γ (H), IL-6 (I), and IL-5 (J). N = 5 mice per group; *$p < 0.05$, ****$p < 0.0001$ as indicated by one-way ANOVA with Tukey's post hoc test. WT, wild type.

expression *in vitro* and on the immune response to the vaccine material itself, as well as on the downstream adaptive response to the encoded antigen. Exchanging uridine with m1Ψ and cleaning up synthesis by-products significantly increased the anti-hemagglutinin antibody titer compared with *in vitro* transcribed mRNA with canonical nucleotides. Exploring the early transcriptomic response in the lymph node draining the injected muscle, we observed a significant up-regulation in genes that might dampen the response to an mRNA vaccine including *RnaseL*, *Ctla4*, and *Stat1*. The D1-uRNA mRNA induced significantly higher levels of inflammatory cytokines in the sera at 6 and 24 h after immunization. To test the efficacy of the different vaccines, mice were infected with influenza virus; the cC1-modRNA-vaccinated mice were significantly more protected against disease than the D1-uRNA-immunized group. Interestingly there was a significant difference in the antibody response between the cC1-modRNA and D1-modRNA groups, but no significant difference in the transcriptomic signatures. This may reflect the *in vitro* expression, where we saw that dsRNA removal and Cap1 incorporation improved HA expression in HEK293T cells.

Our hypothesis was that sensing of the different mRNA vaccines by the innate immune system would have an impact on the downstream adaptive immune response. To investigate this, we compared the response at the transcriptome level in the draining lymph node of the injected

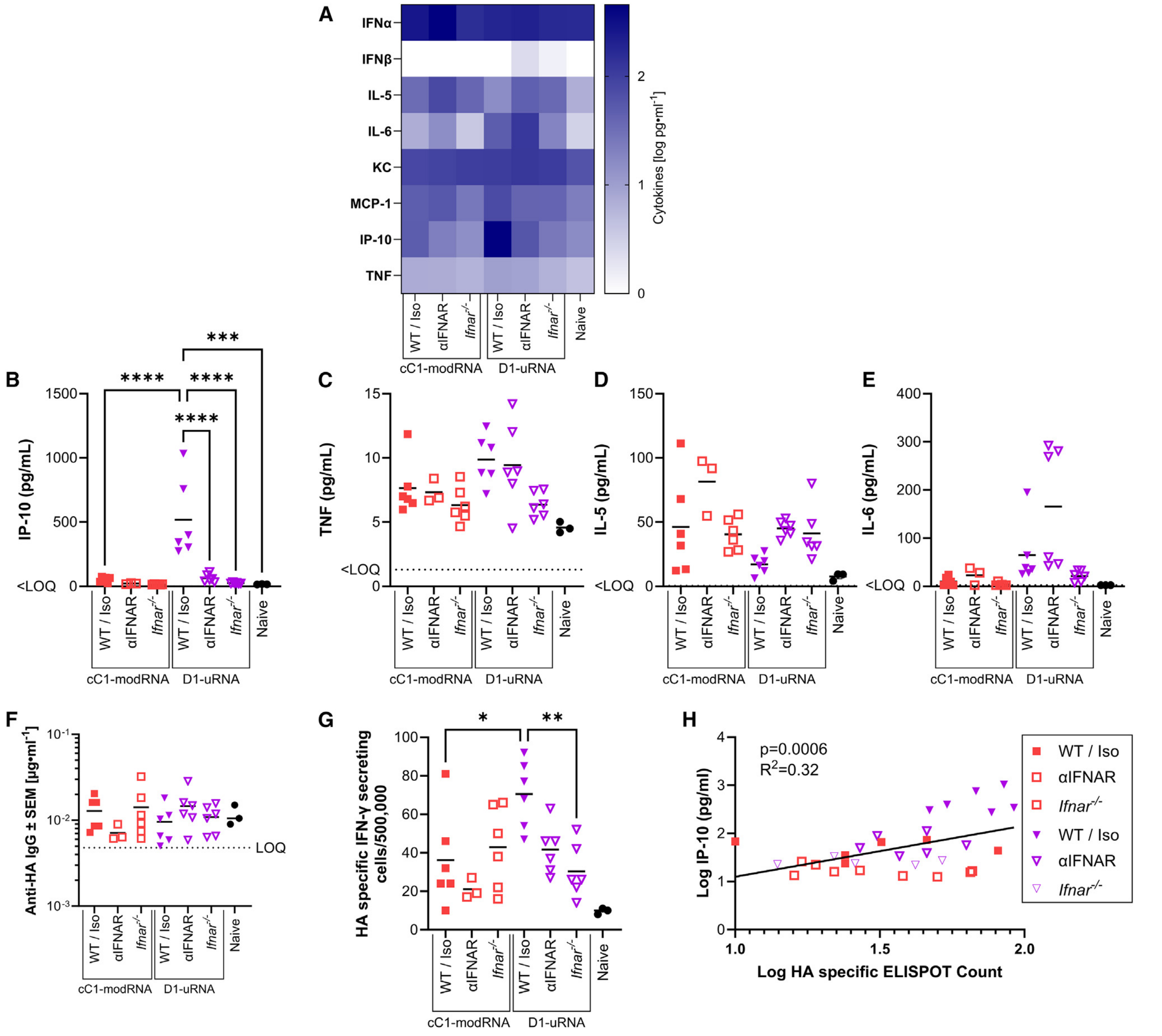

Figure 6. Blocking IFN signaling alters the inflammatory profile after immunization and the T cell response

(A–G) C57BL/6 mice treated with IFNAR blocking antibody (αIFNAR: 1 mg MAR1 intraperitoneally 24 h before immunization), or an isotype control (wild type [WT]/Iso), or *Ifnar*$^{-/-}$ mice were immunized intramuscularly with 1 μg cC1-modRNA or D1-uRNA at 0 and 28 days responses compared with a no vaccine control (naive); 6 h after immunization sera were collected to measure cytokines by MSD (A): individual cytokines IP-10 (B), TNF (C), IL5 (D), and IL-6 (E). At 14 days after the second dose, mice were culled and sera collected for anti-HA IgG (F) and spleens for HA specific ELISPOT (G). IP-10 24 h after immunization was compared HA-specific ELISPOT responses (H). N = 6 per group, except cC1-modRNA and control where N = 3. *p < 0.05, **p < 0.001, ***p < 0.001, ****p < 0.0001 as indicated by one-way ANOVA with Tukey's post hoc test.

muscle. Previous studies have investigated the transcriptomic response to m1Ψ modified mRNA vaccines: in the draining lymph nodes of non-human primates, there was a significant up-regulation of STAT1 and inflammatory cytokine genes at 24 h after immunization.[23] Cross platform comparisons have been made between mice and a human tissue model using *in vitro*-transcribed mRNA with canonical nucleosides, detecting a significant up-regulation of IFN-inducible genes and inflammatory cytokines in the skin and lymph nodes.[24] However, a comparison has not been made between different mRNA vaccine formats before. Here, we observed a significant up-regulation of multiple genes after D1-uRNA immunization, but not after cC1-modRNA or D1-modRNA. At the transcriptional module level, in our study we detected up-regulation of similar pathways to previous studies after vaccination with BNT162b2, an m1Ψ containing mRNA vaccine.[19] Of the genes that were differentially up-regulated in the lymph nodes after D1-uRNA vaccine, there were some that may functionally contribute to the difference

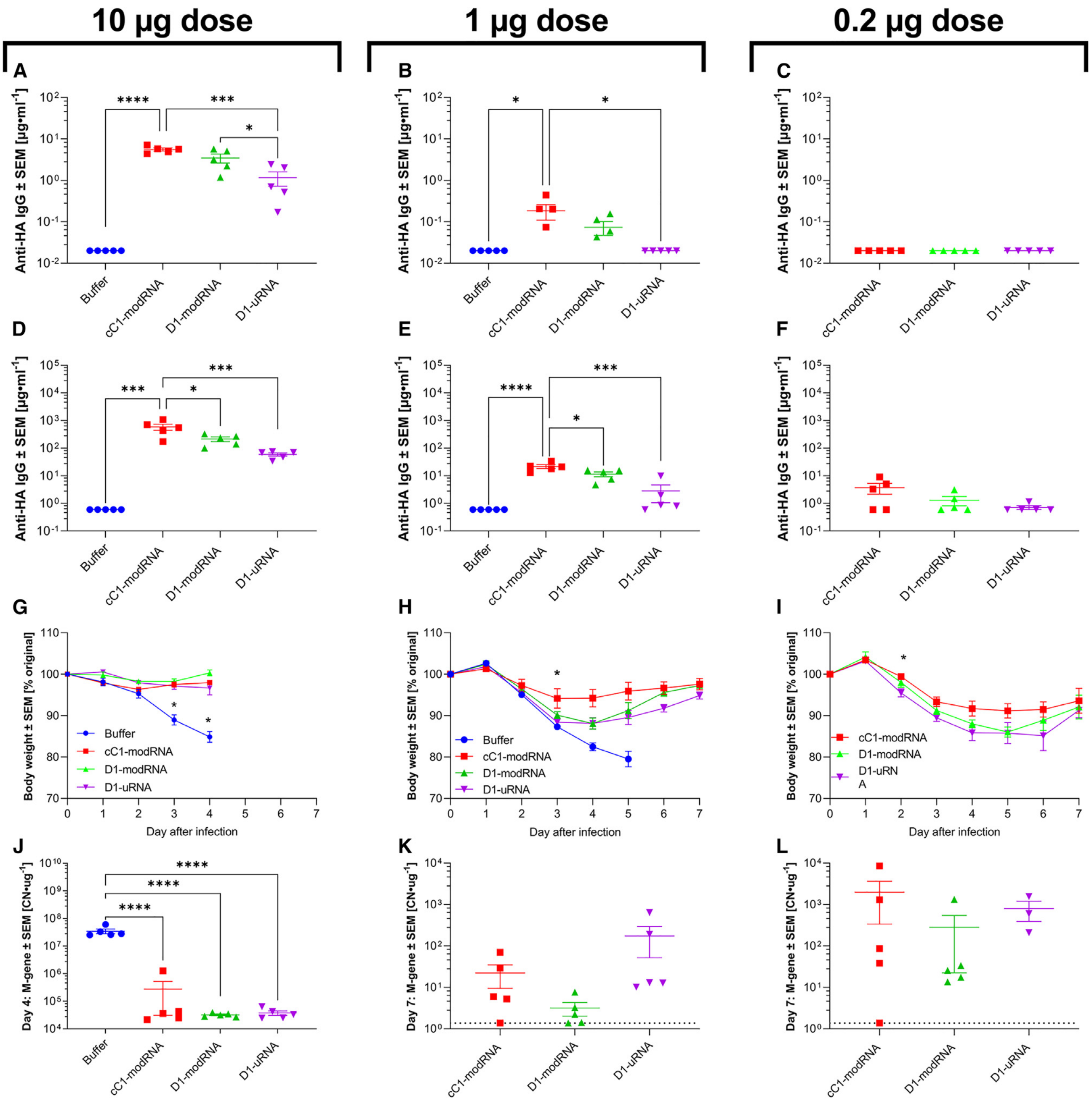

Figure 7. Differences in induced antibody levels between different mRNA vaccine formulations

(A–L) BALB/c mice were immunized intramuscularly at day 0 and days 21 with 10, 1, or 0.2 μg mRNA expressing HA from H1 influenza; the mRNA was either D1-uRNA, D1-modRNA, or cC1-modRNA. Blood was collected to measure anti-HA antibody responses at 4 (A– C) and 8 weeks (D–F). Mice were infected intranasally with influenza virus at 8 weeks, weight loss was measured after infection (G–I). Viral load after infection was measured by absolute RT-qPCR quantification taken on day 4 (J) or day 7 (K, L), copy number (CN) per microgram RNA extracted from lung tissue is shown. Dotted line represents lower limit of detection. N = 5 mice per group; *$p < 0.05$, ***$p < 0.001$, ****$p < 0.0001$ as indicated by one-way ANOVA with Tukey's post hoc test. In (G–I) * vs. buffer control.

in response. One of the most striking genes was *RnaseL* (formerly 2-5A-dependent RNase); this is an extremely well characterized IFN-stimulated gene (ISG), first discovered in the 1970s[25] and cloned in the 1990s.[26] RNaseL breaks down single-stranded RNA and, therefore, is likely to play an important role in dampening expression from an mRNA vaccine.[27] It has been demonstrated that pseudouridine-containing RNA is resistant to cleavage by RNaseL.[28] RNaseL is activated by 5′-phosphorylated, 2′-5′-linked oligoadenylates known as 2-5A,

which are normally generated by members of the OAS family. We did not observe up-regulation of OAS genes, but both OAS and RNaseL are ISGs and we observed significantly increased levels of STAT which acts downstream of the IFN receptor to induce transcription of ISG. Another interesting observation in the transcriptomic dataset was a significant increase in many genes from the P200 family, which are associated with cell differentiation and proliferation[29]; what roles they may play in the immune response to RNA vaccination needs further investigation. It was notable that there was no detectable gene signature for either the cC1-modRNA or D1-modRNA groups relative to the control; this may reflect a different kinetic as seen in the cytokine data, where the responses in these groups were later than the D1-uRNA. It may also reflect that the sequencing was performed on lymph nodes, and there might be a different profile in the muscle.

As well as differential response in genes after immunization between cC1-modRNA, D1-modRNA and D1-uRNA, there was a difference in systemic cytokines. The kinetic of the response to the D1-uRNA was different to the other two vaccines, with significantly higher levels of a wide range of cytokines and chemokines; although these may be beneficial in terms of recruiting cells necessary for the adaptive immune response, they may also lead to more rapid clearance of the transgene.[30] A greater cytokine response to D1-uRNA than m1Ψ-containing RNA was seen in the initial studies developing the platform.[5] There was also a cytokine response at 6 and 24 h after immunization, and detectable levels of some cytokines in all groups; cytokine responses in the serum have also been detected 24 h after immunization in BNT162b2 mRNA vaccine recipients.[31] Other studies have shown a rapid and sustained cytokine response to mRNA vaccines in mice,[32] with similar elevations in IL-5, IL-6, and MCP-1.[33] There is likely a balance between vaccine-induced inflammation leading to the recruitment of the cells necessary for an adaptive immune response and suppression of vaccine transgene expression. The role of individual cytokines in the adaptive immune response to mRNA vaccines needs further investigation. A recent study using a lipid nanoparticle (LNP)-formulated mRNA vaccine demonstrated that vaccine induced IL-6 plays a role in the induction of T follicular helper cells and germinal center B cells.[34] Other studies have observed that m1Ψ-modified RNA can induce high levels of Tfh and GC B cells.[35] We saw a correlation between cytokines and the antibody response to the vaccine—increased TNF was associated with lower antibody responses, which suggests that inflammation induced by D1-uRNA RNA does dampen the downstream response. Interestingly, we saw a positive correlation between IL-5 and the adaptive immune response; we have previously observed this to an MF59 formulated influenza vaccine.[36] IL-5 has a role in the development of B cells.[37] Interestingly, we saw that blocking IFNAR signaling altered the cytokine response to vaccination; IFNAR blockade increased IL-6 and IL-5 responses to D1-uRNA. Interestingly, IFNAR blockade significantly reduced IP-10 responses and reduced T cell responses to the D1-uRNA immunization; this reflected a recent cancer study where anti-IFNAR treatment of unmodified RNA vaccination significantly reduced CD8 T cell responses.[38] This suggests that different approaches may be needed, depending on the type of adaptive response required. One caveat is that the studies in *Ifnar*$^{-/-}$ and IFNAR blockade were performed in C57BL/6 mice, rather than BALB/c, which was used for the rest of the studies (because of background strain). We saw a much greater response to the D1-uRNA in the C57BL/6 mice than cC1-modRNA, which was different from the BALB/c. There are well characterized differences between these inbred strains in many different infection and vaccination settings; a recent study using LNP-encapsulated, unmodified RNA saw no antibody but a substantial T cell response in C57BL/6 mice, compared with a mixed antibody and T cell response in BALB/c.[39] These strain differences may help to provide further insight into the mechanism of the induction of immune responses to RNA vaccines.

We also investigated the cells in the muscle (the injection site) and the draining lymph node. There was an increase in dendritic cells in the lymph node of D1-uRNA-immunised mice, and this reflected the observation that the lymph nodes in these mice were enlarged compared with the other groups, which may indicate a more rapid kinetic of response. There was a much greater percentage of Ly6G$^+$ (neutrophil) cells in the lymph nodes after immunization with all three mRNA vaccine types; neutrophils have been observed as being able to shuttle antigen to lymph nodes.[40] In mice, neutrophils are recruited by CXCL1 (KC), among other chemokines, and we observed significantly elevated KC levels in all vaccinated animals compared with the buffer control. Interestingly there was a difference at the transcription level of the chemokine receptors used by neutrophils—the D1-uRNA group had significantly higher levels of *Cxcr1* and *Ackr1*, whereas the cC1-modRNA group had elevated levels of *Cxcr2*. What this means in terms of the functional response is unclear; ACKR1 has a role in neutrophil extravasation, while CXCR1 and CXCR 2 alter cell function.[41]

Overall, we observed that the incorporation of m1Ψ led to a significant increase in the antibody response compared with unmodified RNA, which correlated with key transcriptional changes in RNA-sensing and IFN-related genes. Even though the combination of Cap1 and dsRNA removal (cC1-modRNA) did not show significant transcription changes compared with control; it led to the best antibody response and best protection against disease after influenza virus infection among the tested RNA formats. Moreover, the differences between the three different mRNA vaccine formats may be underestimated in the murine model, as mice tend to be less sensitive to stimuli of the innate immunity.[42] Testing in other mammal species might be required to better assess the impact on innate and adaptive immunity of different mRNA vaccines designs. Nevertheless, it is clear that the incorporation of m1Ψ and Cap1 into mRNA vaccines has led to their considerable success during the COVID-19 pandemic.[2] There are a number of limitations for comparing the efficacy data of different mRNA formats used for vaccination in clinical trials, as the vaccines were not compared head-to-head. However, it is of note that the vaccine efficacy of CVnCoV (48.2%)[43] was lower than BNT162b2 (95%)[44] or mRNA-1273 (94%).[45] CVnCoV is exclusively composed of canonical nucleosides,[46] whereas BNT162b2 and mRNA-1273 both incorporate m1Ψ, which suggests there is clinical benefit to silencing the immunoreactivity of mRNA.

MATERIALS AND METHODS

RNA synthesis by *in vitro* transcription

The influenza virus HA sequence was obtained from the Influenza A strain A/California/07/2009 (GenBank: FJ981613.1). DNA templates were cloned into a plasmid vector with backbone sequence elements (T7 promoter), 5′ and 3′ UTR, and a 100-nucleotide poly(A) tail interrupted by a linker (A30LA70, where L = GCAUAUGACU) for improved RNA stability and translational efficiency.[47,48] The DNA was purified, quantified using spectrophotometry, and *in vitro* transcribed with T7 RNA polymerase (MEGAscript T7 Transcription Kit, Thermo Fisher, formerly Ambion). The general procedure was carried out similarly as described before[49] starting with linear DNA template containing the T7 promoter.

The D1-uRNA was produced in the presence of a β-S-ARCA (D1) cap analogue, as described previously.[47,50,51] The D1-modRNA was also produced in the presence of a β-S-ARCA(D1) cap analogue, but with m1Ψ-5′-triphosphate (m1ΨTP; ThermoFisher Scientific) replacing uridine-5′-triphosphate (UTP).[52] The Cap1-modRNA (cC1-modRNA) was produced in the presence of a trinucleotide cap1 analogue ((m_2 7,3′-O)Gppp(m2′-O)ApG) (TriLink) and with m1ΨTP replacing UTP. For cC1-modRNA a final cellulose purification step was included to reduce the dsRNA content.[15] All RNAs were purified using magnetic particles.[53] Concentration and quality were assessed by microfluidic capillary electrophoresis and spectrophotometry (2100 Bioanalyzer, Agilent).

Production of LNPs

LNPs were manufactured by controlled mixing of RNA dissolved in aqueous buffer with an ethanolic solution of lipids using a NanoAssemblr (Precision Nanosystems). The resulting aqueous-organic dispersion of LNPs was subjected to dialysis against water for removal of ethanol. The LNP contains the RNA, an ionizable lipid (N,N-dimethyl-2,3-bis[(Z)-octadec-9-enoxy]propan-1-amine), a PEGylated lipid, (2-[(polyethylene glycol)-2000]-N,N-ditetradecylacetamide), and two structural lipids (1,2-distearoyl-sn-glycero-3-phosphocholine) and cholesterol). LNPs were produced and shipped at 4°C for direct *in vivo* application.

LNP characterization

Average LNP hydrodynamic size (*Z*-average in nm) and polydispersity index were determined by dynamic light scattering on a DynaPro PlateReader II and analyzed with the Dynamics v.7.8.1 software (both from Wyatt Technology). LNP samples were diluted to 0.01 mg/mL in PBS. All samples were measured in duplicate, ten data points were recorded per replicate, each measurement lasted 10s.

Cell culture

HEK293T (ATCC) and MEF (ATCC) were cultured in DMEM (Gibco) supplemented with 10% fetal bovine serum (Sigma-Aldrich) and GlutaMax (Gibco). Cell lines were tested for mycoplasma contamination after receipt, before expansion and cryopreservation. HEK293T cells and MEF cells were not used above 25 and 15 passages, respectively.

Transfection

We plated 2×10^5 cells per well in a 12-well plate and transfected after attachment with either 80 or 150 ng RNA on the same day. Transfection reactions were prepared in Opti-MEM (Gibco) at a concentration of 1 ng/μL, including 1 μL RiboJuice Boost Reagent and RiboJuice Transfection Reagent per microgram RNA (Merck Millipore). Transfection reaction was added to the cells within 5 min of preparation and harvest 18 h later as described in the flow cytometry for *in vitro* experiments section.

Flow cytometry for *in vitro* experiments

Transfected HEK 293T or MEF cells were detached from the cell culture dish 18 h post transfection by adding 230 μL Accutase (Gibco) for 10 min. Then cells were stained with a Fixable Viability Dye (eBioscience). To detect cell-surface antigens, cells were stained with anti-HA antibody M175 (Takara, dilution 1:175) for 30 min on ice. After two washes with PBS-1% BSA, cells were stained with anti-mouse antibody labeled with AF488 (Dianova, dilution 1:100). After two washes with PBS-1% BSA, cells were fixed for 10 min at room temperature (Fixation Buffer, BioLegend). Cells were acquired on a FACSCelesta flow cytometer (BD Biosciences) using BD FACSDiva software version 8.0.1 and analyzed with FlowJo software version 10.6.2 (FlowJo, BD Biosciences).

Mouse immunization and infection

For immunogenicity and challenge studies, 6- to 8-week-old female BALB/c mice were obtained from Charles River Ltd (Beckenham, UK) and kept in specific-pathogen-free conditions in accordance with United Kingdom's Home Office guidelines. All work was approved by the Animal Welfare and Ethical Review Board at Imperial College London under PPL P4EE85DED. Mice were immunized with 50 μL RNA vaccine or buffer control in the vastus lateralis muscle (i.m.). Immunization was either a single dose for acute measurements of the innate immune response or a prime-boost regime with 21 days between doses.

In the study looking at the role of IFNAR, C57BL6 mice (Charles River Laboratories) and IFNAR$^{-/-}$ (from Jackson Laboratory: B6.129S2-Ifnar1^{tm1Agt}/Mmjax) were used. At 24 h before immunization, C57BL/6 mice were intraperitoneally treated with IFNAR-blocking antibody (αIFNAR: 1 mg MAR1; Assay Genie) or control antibody. In this study, mice were immunized with 1 μg RNA i.m. with a 21-day interval between prime and boost. Mice were culled 21 days after boost.

For infections, mice were anesthetized using isoflurane followed by i.n. application with 3×10^4 pfu A/California/7/2009 (H1N1) influenza virus.[54] Virus was grown in MDCK cells, in serum-free DMEM supplemented with 1 μg/mL trypsin and virus titer was determined by plaque assay.

Tissue and cell recovery and isolation

At specified time points after immunization, blood samples were taken by tail vein bleed and sera isolated after clotting by

centrifugation. Mice were culled using 100 μL intraperitoneal pentobarbitone (20 mg dose, Pentoject, Animalcare Ltd.) and lung tissue collected as previously described.[55] At the terminal bleed, blood was collected from carotid vessels and sera isolated after clotting by centrifugation. Where used, spleens were collected at day 42 and processed as previously described.[56]

Single-cell suspensions from vastus muscle were prepared by digestion in 1 mL Liberace (12.5 μg/mL, Roche), DNase (200 μg/mL, Sigma), and hyaluronidase (50 μg/mL, Molecular Dimensions), with shaking for 1 h at 37°C before passing through 100-μm cell strainers, then recovered after centrifugation at 500×*g* for 5 min[57] For lymph node single-cell suspensions, we followed a similar protocol, the exception being the digestion step, which was done in DNase (200 μg/mL) for 15 min before being homogenized by passage through 100-μm cell strainers, then centrifuged at 500×*g* for 5 min.

Supernatants were discarded and the cell pellet treated with red blood cell lysis buffer (ACK; 0.15 M ammonium chloride, 1 M potassium hydrogen carbonate, and 0.01 mM EDTA, pH 7.2) before centrifugation at 200×*g* for 5 min. Cells were resuspended in RPMI 1640 medium with 10% fetal calf serum, and viable cell numbers determined by trypan blue exclusion.

RNA sequencing

RNA was extracted by the Trizol method after tissue disruption using a TissueLyzer (Qiagen). RNA concentrations were determined using a Nanodrop (Thermo Fisher Scientific) before sending for sequencing to Imperial BRC Genomics Facility sequencing facility. RNA quality check and library preparation was performed by Imperial College London using the Illumina PE150 at a target depth of 50 million 100–150 bp-end reads per sample. The obtained raw RNA sequencing data files were first checked with FastQC (v0.11.9) to ensure good quality scores, GC content, and no adaptor reads, then appropriate adjustment was made using the program Trimmomatic (v1.0.40).[58] Raw reads were then mapped to Mouse Reference Genome (Gmc38) using STAR (Spliced Transcripts Alignment to a Reference, v6.2.0)[59] and count data for each gene was performed using featureCounts (v1.22.2).[60] For genes with multiple isoforms, only the transcript with the highest mean expression between all samples was selected. Raw and processed data are available in the X database. Genes with counts across more than two samples were excluded and the data were normalized using rlog function from the Limma Bioconductor package.[61]

Multiplex cytokine measurements

Cytokines in blood were assessed using custom mouse kits from Meso Scale Discovery as a 10-spot U-PLEX (K15069L-2), including the following analytes (lower limit of detection in pg/mL in parentheses): GM-CSF (0.16), IFN-γ (0.16), IL-5 (0.63), IL-6 (4.8), IP-10 (0.5), KC (0.43), MCP-1 (1.4), MIP-1α (0.21), MIP-1β (13), and TNF (1.3).

Flow cytometry for *ex vivo* experiments

Live muscle or lymph node cells were added to wells of a U-bottom 96-well plate then spun at 2,000 rpm for 2 min at 4°C to remove the supernatant. Cells were incubated for 20 min at 4°C in the dark with 100 μL Live/Dead violet dye (Thermo Fisher Scientific, L34955), then centrifuged at 2,000 rpm for 2 min and the supernatant was discarded. The cell pellet was resuspended in Fc block (Anti-CD16, BD, Cat.no.: 6266549) in PBS-1% BSA and stained with the following surface antibodies Ly6G (7046845 BD), CD11c (6209870, BD), F4/80 (15-4801-82, eBioscience), and MHCII (4289686, eBioscience) for 1 h in the dark. The excess antibodies were washed off with 1% BSA in PBS three times before samples were filtered through FAC tubes. Cells were acquired on an LSR Fortessa Flow cytometer (BD Biosciences) and analyzed with the FlowJo software (BD Biosciences). Fluorescence minus one controls were used for surface stains. The gating strategy is shown in Figure S3.

Semi-quantitative antigen-specific ELISA

Antibodies specific to influenza H1N1 virus were measured in sera using a standardized ELISA. MaxiSorp 96-well plates (Nunc) were coated with 1 μg/mL A/California/07/2009 HA (His-tagged HA; 11085-V08B, SinoBiological) surface protein or a combination of anti-murine lambda and kappa light chain specific antibodies (AbD Serotec) and incubated overnight at 4°C. Plates were blocked with 1% BSA in PBS. Bound IgG was detected using horseradish peroxidase-conjugated goat anti-mouse IgG (AbD Serotec) and the tetramethylbenzidine substrate, followed by H_2SO_4 as stop solution. The resulting optical density was read at 450 nm. Alternatively, IgG1 or IgG2a were detected using subtype-specific secondary antibodies. A dilution series of recombinant murine immunoglobulin was used as a standard to quantify specific antibodies.

HAI

All samples were analyzed by HAI assay using A/California/7/2009 (H1N1) virus strain as described.[62] Serum samples were pre-treated with receptor-destroying enzyme (RDE; Denka Seiken) for 18 h at 37°C before inactivating the enzyme at 56°C for 1 h. RDE-treated serum was 2-fold serially diluted across the plate with PBS and incubated with pre-diluted four hemagglutinating units of virus per well for 30 min at room temperature. We then added 50 μL of 1% turkey erythrocytes diluted in PBS to each well, and the plate was incubated for 30 min at 4°C before scoring the response.

T cell ELISpot

IFN-γ ELISpot assays were performed on mouse splenocytes using a commercial kit (cat.no. ab64029, AbCam) following the manufacturer's recommendations. Cells were stimulated with 2 μg/mL anti-CD28 (Clone 37.51: BD) and 15-mer sequences with 11 amino acids overlap peptides from influenza A (H1N1) HA (Peptivator; Miltenyi Biotech). The spots were counted using the AID iSpot reader (AID) and ELISpot Reader software V 7.0.

Influenza viral load

Viral load *in vivo* was assessed by Trizol extraction of RNA from frozen lung tissue disrupted in a TissueLyzer (Qiagen, Manchester, UK). RNA was reverse-transcribed into cDNA and quantitative RT-PCR for influenza M was carried out using 0.1 μM forward

primer (5′-AAGACAAGACCAATYCTGTCACCTCT-3′), 0.1 μM reverse primer (5′-TCTACGYTGCAGTCCYCGCT-3′), and 0.2 μM probe (5′-FAM-TYACGCTCACCGTGCCCAGTG-TAMRA-3′) on a Stratagene Mx3005p system (Agilent Technologies). An influenza M gene plasmid was used as standard to determine M-specific RNA copy number.

Statistical analysis

Statistical analyses listed in the figure legends were performed using GraphPad Prism 9 (GraphPad Software Inc.). Multiple correlations were performed by Pearson r and individual correlations by simple linear regression. Differential gene expression was carried out using Limma package (v3.41.15). PCA-normalized sequence data were analyzed using pcaExplorer (v1.0.2).[63] A gene was considered differentially expressed if the adjusted p value was <0.05. Gene set enrichment analysis was performed with R package tmod (version 0.34) using CERNO statistical test.[64] Network analysis of significant genes was performed using InnateDB (https://www.innatedb.com) before submitting to NetworkAnalyst (v10.0).

DATA AND CODE AVAILABILITY

RNA data are on Arrayexpress accession number E-MTAB-13194. Material sharing would be subject to MTA.

SUPPLEMENTAL INFORMATION

Supplemental information can be found online at https://doi.org/10.1016/j.omtn.2023.102045.

ACKNOWLEDGMENTS

The authors thank Kasia S., Paul McKay, Hadi Sallah, Sophie Higham, and Catherine U. for assistance in the *in vivo* studies and Lesley Rawlinson for laboratory management. Thanks to A. Murray and J. Holland for interesting discussion. For the purpose of open access, the author has applied a Creative Commons Attribution (CC BY) license to any Author Accepted Manuscript version arising.

This study was supported by EU FP7 Health (ADITEC). Data presented were generated in a collaboration started in 2017.

AUTHOR CONTRIBUTIONS

Z.W.: data curation, formal analysis, visualization, and investigation; E.J.: investigation, formal analysis, and writing - review and editing; D.S.: investigation; S.K.: resources, writing - review and editing; R.C.: investigation; K.E.F.: investigation; J.M.:, resources; C.S.: investigation; L.M.: investigation; L.S.: formal analysis, writing—review and editing; A.B.V.: funding acquisition and investigation; K.K.: conceptualization; U.S.: conceptualization; S.E.: conceptualization, resources, writing—review and editing; J.S.T.: conceptualization, funding acquisition, Investigation, and writing—original draft.

DECLARATION OF INTERESTS

E.J.J., S.K., J.M., L.A.S., A.B.V., K.K., U.S., and S.E. are employees at BioNTech SE (Mainz, Germany). U.S. is cofounder and management board member of BioNTech SE (Mainz, Germany). A.B.V., K.K., U.S., and S.E. are inventors on patents and patent applications related to RNA technology. A.B.V., K.K., U.S., and S.E. hold securities from BioNTech SE.

REFERENCES

1. Tregoning, J.S., Brown, E.S., Cheeseman, H.M., Flight, K.E., Higham, S.L., Lemm, N.M., Pierce, B.F., Stirling, D.C., Wang, Z., and Pollock, K.M. (2020). Vaccines for COVID-19. Clin. Exp. Immunol. *202*, 162–192.
2. Tregoning, J.S., Flight, K.E., Higham, S.L., Wang, Z., and Pierce, B.F. (2021). Progress of the COVID-19 vaccine effort: viruses, vaccines and variants versus efficacy, effectiveness and escape. Nat. Rev. Immunol. *21*, 626–636.
3. Sahin, U., Muik, A., Vogler, I., Derhovanessian, E., Kranz, L.M., Vormehr, M., Quandt, J., Bidmon, N., Ulges, A., Baum, A., et al. (2021). BNT162b2 vaccine induces neutralizing antibodies and poly-specific T cells in humans. Nature *595*, 572–577.
4. Baden, L.R., El Sahly, H.M., Essink, B., Kotloff, K., Frey, S., Novak, R., Diemert, D., Spector, S.A., Rouphael, N., Creech, C.B., et al. (2021). Efficacy and Safety of the mRNA-1273 SARS-CoV-2 Vaccine. N. Engl. J. Med. Overseas. Ed. *384*, 403–416.
5. Karikó, K., Buckstein, M., Ni, H., and Weissman, D. (2005). Suppression of RNA recognition by Toll-like receptors: the impact of nucleoside modification and the evolutionary origin of RNA. Immunity *23*, 165–175.
6. Laczkó, D., Hogan, M.J., Toulmin, S.A., Hicks, P., Lederer, K., Gaudette, B.T., Castaño, D., Amanat, F., Muramatsu, H., Oguin, T.H., 3rd, et al. (2020). A Single Immunization with Nucleoside-Modified mRNA Vaccines Elicits Strong Cellular and Humoral Immune Responses against SARS-CoV-2 in Mice. Immunity *53*, 724–732.e727.
7. Pardi, N., Parkhouse, K., Kirkpatrick, E., McMahon, M., Zost, S.J., Mui, B.L., Tam, Y.K., Karikó, K., Barbosa, C.J., Madden, T.D., et al. (2018). Nucleoside-modified mRNA immunization elicits influenza virus hemagglutinin stalk-specific antibodies. Nat. Commun. *9*, 3361.
8. Pardi, N., Hogan, M.J., Pelc, R.S., Muramatsu, H., Andersen, H., DeMaso, C.R., Dowd, K.A., Sutherland, L.L., Scearce, R.M., Parks, R., et al. (2017). Zika virus protection by a single low-dose nucleoside-modified mRNA vaccination. Nature *543*, 248–251.
9. Hotz, C., Wagenaar, T.R., Gieseke, F., Bangari, D.S., Callahan, M., Cao, H., Diekmann, J., Diken, M., Grunwitz, C., Hebert, A., et al. (2021). Local delivery of mRNA-encoded cytokines promotes antitumor immunity and tumor eradication across multiple preclinical tumor models. Sci. Transl. Med. *13*, eabc7804.
10. Krienke, C., Kolb, L., Diken, E., Streuber, M., Kirchhoff, S., Bukur, T., Akilli-Öztürk, Ö., Kranz, L.M., Berger, H., Petschenka, J., et al. (2021). A noninflammatory mRNA vaccine for treatment of experimental autoimmune encephalomyelitis. Science *371*, 145–153.
11. Schlee, M., and Hartmann, G. (2016). Discriminating self from non-self in nucleic acid sensing. Nat. Rev. Immunol. *16*, 566–580.
12. Karikó, K., Muramatsu, H., Welsh, F.A., Ludwig, J., Kato, H., Akira, S., and Weissman, D. (2008). Incorporation of Pseudouridine Into mRNA Yields Superior Nonimmunogenic Vector With Increased Translational Capacity and Biological Stability. Mol. Ther. *16*, 1833–1840.
13. Kuhn, A.N., Diken, M., Kreiter, S., Selmi, A., Kowalska, J., Jemielity, J., Darzynkiewicz, E., Huber, C., Türeci, O., and Sahin, U. (2010). Phosphorothioate cap analogs increase stability and translational efficiency of RNA vaccines in immature dendritic cells and induce superior immune responses in vivo. Gene Ther. *17*, 961–971.
14. Ramanathan, A., Robb, G.B., and Chan, S.H. (2016). mRNA capping: biological functions and applications. Nucleic Acids Res. *44*, 7511–7526.
15. Baiersdörfer, M., Boros, G., Muramatsu, H., Mahiny, A., Vlatkovic, I., Sahin, U., and Karikó, K. (2019). A Facile Method for the Removal of dsRNA Contaminant from In Vitro-Transcribed mRNA. Mol. Ther. Nucleic Acids *15*, 26–35.
16. Alexopoulou, L., Holt, A.C., Medzhitov, R., and Flavell, R.A. (2001). Recognition of double-stranded RNA and activation of NF-κB by Toll-like receptor 3. Nature *413*, 732–738.
17. Maruggi, G, Ulmer, JB, Rappuoli, R, and Yu, D. Self-amplifying mRNA-Based Vaccine Technology and its Mode of Action. Springer Berlin. pp 1-40.

18. Schoggins, J.W. (2019). Interferon-Stimulated Genes: What Do They All Do? Annu. Rev. Virol. *6*, 567–584.
19. Arunachalam, P.S., Scott, M.K.D., Hagan, T., Li, C., Feng, Y., Wimmers, F., Grigoryan, L., Trisal, M., Edara, V.V., Lai, L., et al. (2021). Systems vaccinology of the BNT162b2 mRNA vaccine in humans. Nature *596*, 410–416.
20. Kauffman, K.J., Mir, F.F., Jhunjhunwala, S., Kaczmarek, J.C., Hurtado, J.E., Yang, J.H., Webber, M.J., Kowalski, P.S., Heartlein, M.W., DeRosa, F., and Anderson, D.G. (2016). Efficacy and immunogenicity of unmodified and pseudouridine-modified mRNA delivered systemically with lipid nanoparticles in vivo. Biomaterials *109*, 78–87.
21. Chaussabel, D., Quinn, C., Shen, J., Patel, P., Glaser, C., Baldwin, N., Stichweh, D., Blankenship, D., Li, L., Munagala, I., et al. (2008). A modular analysis framework for blood genomics studies: application to systemic lupus erythematosus. Immunity *29*, 150–164.
22. Li, S., Rouphael, N., Duraisingham, S., Romero-Steiner, S., Presnell, S., Davis, C., Schmidt, D.S., Johnson, S.E., Milton, A., Rajam, G., et al. (2014). Molecular signatures of antibody responses derived from a systems biology study of five human vaccines. Nat. Immunol. *15*, 195–204.
23. Liang, F., Lindgren, G., Lin, A., Thompson, E.A., Ols, S., Röhss, J., John, S., Hassett, K., Yuzhakov, O., Bahl, K., et al. (2017). Efficient Targeting and Activation of Antigen-Presenting Cells In Vivo after Modified mRNA Vaccine Administration in Rhesus Macaques. Mol. Ther. *25*, 2635–2647.
24. Edwards, D.K., Jasny, E., Yoon, H., Horscroft, N., Schanen, B., Geter, T., Fotin-Mleczek, M., Petsch, B., and Wittman, V. (2017). Adjuvant effects of a sequence-engineered mRNA vaccine: translational profiling demonstrates similar human and murine innate response. J. Transl. Med. *15*, 1.
25. Clemens, M.J., and Williams, B.R. (1978). Inhibition of cell-free protein synthesis by pppA2'p5'A2'p5'A: a novel oligonucleotide synthesized by interferon-treated L cell extracts. Cell *13*, 565–572.
26. Zhou, A., Hassel, B.A., and Silverman, R.H. (1993). Expression cloning of 2-5A-dependent RNAase: a uniquely regulated mediator of interferon action. Cell *72*, 753–765.
27. Liang, S.L., Quirk, D., and Zhou, A. (2006). RNase L: its biological roles and regulation. IUBMB Life *58*, 508–514.
28. Anderson, B.R., Muramatsu, H., Jha, B.K., Silverman, R.H., Weissman, D., and Karikó, K. (2011). Nucleoside modifications in RNA limit activation of 2'-5'-oligoadenylate synthetase and increase resistance to cleavage by RNase L. Nucleic Acids Res. *39*, 9329–9338.
29. Zhao, H., Gonzalezgugel, E., Cheng, L., Richbourgh, B., Nie, L., and Liu, C. (2015). The roles of interferon-inducible p200 family members IFI16 and p204 in innate immune responses, cell differentiation and proliferation. Genes Dis. *2*, 46–56.
30. Mann, J.F.S., McKay, P.F., Klein, K., Pankrac, J., Tregoning, J.S., and Shattock, R.J. (2021). Blocking T-cell egress with FTY720 extends DNA vaccine expression but reduces immunogenicity. Immunology *165*, 301–311.
31. Bergamaschi, C., Terpos, E., Rosati, M., Angel, M., Bear, J., Stellas, D., Karaliota, S., Apostolakou, F., Bagratuni, T., Patseas, D., et al. (2021). Systemic IL-15, IFN-γ, and IP-10/CXCL10 signature associated with effective immune response to SARS-CoV-2 in BNT162b2 mRNA vaccine recipients. Cell Rep. *36*, 109504.
32. Lutz, J., Lazzaro, S., Habbeddine, M., Schmidt, K.E., Baumhof, P., Mui, B.L., Tam, Y.K., Madden, T.D., Hope, M.J., Heidenreich, R., and Fotin-Mleczek, M. (2017). Unmodified mRNA in LNPs constitutes a competitive technology for prophylactic vaccines. npj Vaccines *2*, 29.
33. Gebre, M.S., Rauch, S., Roth, N., Gergen, J., Yu, J., Liu, X., Cole, A.C., Mueller, S.O., Petsch, B., and Barouch, D.H. (2021). mRNA Vaccines Induce Rapid Antibody Responses in Mice. Preprint at bioRxiv. https://doi.org/10.1101/2021.11.01.466863.
34. Alameh, M.-G., Tombácz, I., Bettini, E., Lederer, K., Sittplangkoon, C., Wilmore, J.R., Gaudette, B.T., Soliman, O.Y., Pine, M., Hicks, P., et al. (2021). Lipid nanoparticles enhance the efficacy of mRNA and protein subunit vaccines by inducing robust T follicular helper cell and humoral responses. Immunity *54*, 2877–2892.e7.
35. Pardi, N., Hogan, M.J., Naradikian, M.S., Parkhouse, K., Cain, D.W., Jones, L., Moody, M.A., Verkerke, H.P., Myles, A., Willis, E., et al. (2018). Nucleoside-modified mRNA vaccines induce potent T follicular helper and germinal center B cell responses. J. Exp. Med. *215*, 1571–1588.
36. McDonald, J.U., Zhong, Z., Groves, H.T., and Tregoning, J.S. (2017). Inflammatory responses to influenza vaccination at the extremes of age. Immunology *151*, 451–463.
37. Takatsu, K., Kouro, T., and Nagai, Y. (2009). Interleukin 5 in the link between the innate and acquired immune response. Adv. Immunol. *101*, 191–236.
38. Sittplangkoon, C., Alameh, M.G., Weissman, D., Lin, P.J.C., Tam, Y.K., Prompetchara, E., and Palaga, T. (2022). mRNA vaccine with unmodified uridine induces robust type I interferon-dependent anti-tumor immunity in a melanoma model. Front. Immunol. *13*, 983000.
39. Kouji, K., Masaki, I., Nao, J., Misako, N., Kou, H., Kiyoko, I.-H., Seiya, Y., Jun, T., Takako, N., Takashi, S., et al. (2021). Optimization of an LNP-mRNA vaccine candidate targeting SARS-CoV-2 receptor-binding domain. Preprint at bioRxiv. https://doi.org/10.1101/2021.03.04.433852.
40. Calabro, S., Tortoli, M., Baudner, B.C., Pacitto, A., Cortese, M., O'Hagan, D.T., De Gregorio, E., Seubert, A., and Wack, A. (2011). Vaccine adjuvants alum and MF59 induce rapid recruitment of neutrophils and monocytes that participate in antigen transport to draining lymph nodes. Vaccine *29*, 1812–1823.
41. Capucetti, A., Albano, F., and Bonecchi, R. (2020). Multiple Roles for Chemokines in Neutrophil Biology. Front. Immunol. *11*, 1259.
42. Vaure, C., and Liu, Y. (2014). A Comparative Review of Toll-Like Receptor 4 Expression and Functionality in Different Animal Species. Front. Immunol. *5*, 316.
43. Kremsner, P.G., Ahuad Guerrero, R.A., Arana-Arri, E., Aroca Martinez, G.J., Bonten, M., Chandler, R., Corral, G., De Block, E.J.L., Ecker, L., Gabor, J.J., et al. (2021). Efficacy and safety of the CVnCoV SARS-CoV-2 mRNA vaccine candidate in ten countries in Europe and Latin America (HERALD): a randomised, observer-blinded, placebo-controlled, phase 2b/3 trial. Lancet Infect. Dis. *22*, 329–340.
44. Polack, F.P., Thomas, S.J., Kitchin, N., Absalon, J., Gurtman, A., Lockhart, S., Perez, J.L., Pérez Marc, G., Moreira, E.D., Zerbini, C., et al. (2020). Safety and Efficacy of the BNT162b2 mRNA Covid-19 Vaccine. N. Engl. J. Med. *383*, 2603–2615.
45. Baden, L.R., El Sahly, H.M., Essink, B., Kotloff, K., Frey, S., Novak, R., Diemert, D., Spector, S.A., Rouphael, N., Creech, C.B., et al. (2021). Efficacy and Safety of the mRNA-1273 SARS-CoV-2 Vaccine. N. Engl. J. Med. *384*, 403–416.
46. Rauch, S., Roth, N., Schwendt, K., Fotin-Mleczek, M., Mueller, S.O., and Petsch, B. (2021). mRNA-based SARS-CoV-2 vaccine candidate CVnCoV induces high levels of virus-neutralising antibodies and mediates protection in rodents. npj Vaccines *6*, 57.
47. Orlandini von Niessen, A.G., Poleganov, M.A., Rechner, C., Plaschke, A., Kranz, L.M., Fesser, S., Diken, M., Löwer, M., Vallazza, B., Beissert, T., et al. (2019). Improving mRNA-Based Therapeutic Gene Delivery by Expression-Augmenting 3' UTRs Identified by Cellular Library Screening. Mol. Ther. *27*, 824–836.
48. Holtkamp, S., Kreiter, S., Selmi, A., Simon, P., Koslowski, M., Huber, C., Türeci, O., and Sahin, U. (2006). Modification of antigen-encoding RNA increases stability, translational efficacy, and T-cell stimulatory capacity of dendritic cells. Blood *108*, 4009–4017.
49. Vogel, A.B., Kanevsky, I., Che, Y., Swanson, K.A., Muik, A., Vormehr, M., Kranz, L.M., Walzer, K.C., Hein, S., Güler, A., et al. (2021). BNT162b vaccines protect rhesus macaques from SARS-CoV-2. Nature *592*, 283–289.
50. Grudzien, E., Stepinski, J., Jankowska-Anyszka, M., Stolarski, R., Darzynkiewicz, E., and Rhoads, R.E. (2004). Novel cap analogs for in vitro synthesis of mRNAs with high translational efficiency. RNA *10*, 1479–1487.
51. Stepinski, J., Waddell, C., Stolarski, R., Darzynkiewicz, E., and Rhoads, R.E. (2001). Synthesis and properties of mRNAs containing the novel "anti-reverse" cap analogs 7-methyl(3'-O-methyl)GpppG and 7-methyl (3'-deoxy)GpppG. RNA *7*, 1486–1495.
52. Grudzien-Nogalska, E., Kowalska, J., Su, W., Kuhn, A.N., Slepenkov, S.V., Darzynkiewicz, E., Sahin, U., Jemielity, J., and Rhoads, R.E. (2013). Synthetic mRNAs with superior translation and stability properties. Methods Mol. Biol. *969*, 55–72.
53. Berensmeier, S. (2006). Magnetic particles for the separation and purification of nucleic acids. Appl. Microbiol. Biotechnol. *73*, 495–504.
54. Groves, H.T., McDonald, J.U., Langat, P., Kinnear, E., Kellam, P., McCauley, J., Ellis, J., Thompson, C., Elderfield, R., Parker, L., et al. (2018). Mouse Models of Influenza Infection with Circulating Strains to Test Seasonal Vaccine Efficacy. Front. Immunol. *9*, 126.

55. Smith, S.E., Busse, D.C., Binter, S., Weston, S., Diaz Soria, C., Laksono, B.M., Clare, S., Van Nieuwkoop, S., Van den Hoogen, B.G., Clement, M., et al. (2019). Interferon-Induced Transmembrane Protein 1 Restricts Replication of Viruses That Enter Cells via the Plasma Membrane. J. Virol. *93*, e02003-18.

56. Van Roey, G.A., Arias, M.A., Tregoning, J.S., Rowe, G., and Shattock, R.J. (2012). Thymic stromal lymphopoietin (TSLP) acts as a potent mucosal adjuvant for HIV-1 gp140 vaccination in mice. Eur. J. Immunol. *42*, 353–363.

57. Cheeseman, H.M., Carias, A.M., Evans, A.B., Olejniczak, N.J., Ziprin, P., King, D.F.L., Hope, T.J., and Shattock, R.J. (2016). Expression Profile of Human Fc Receptors in Mucosal Tissue: Implications for Antibody-Dependent Cellular Effector Functions Targeting HIV-1 Transmission. PLoS One *11*, e0154656.

58. Bolger, A.M., Lohse, M., and Usadel, B. (2014). Trimmomatic: a flexible trimmer for Illumina sequence data. Bioinformatics *30*, 2114–2120.

59. Dobin, A., Davis, C.A., Schlesinger, F., Drenkow, J., Zaleski, C., Jha, S., Batut, P., Chaisson, M., and Gingeras, T.R. (2013). STAR: ultrafast universal RNA-seq aligner. Bioinformatics *29*, 15–21.

60. Liao, Y., Smyth, G.K., and Shi, W. (2014). featureCounts: an efficient general purpose program for assigning sequence reads to genomic features. Bioinformatics *30*, 923–930.

61. Ritchie, M.E., Phipson, B., Wu, D., Hu, Y., Law, C.W., Shi, W., and Smyth, G.K. (2015). limma powers differential expression analyses for RNA-sequencing and microarray studies. Nucleic Acids Res. *43*, e47.

62. Zhong, Z., Haltalli, M., Holder, B., Rice, T., Donaldson, B., O'Driscoll, M., Le-Doare, K., Kampmann, B., and Tregoning, J.S. (2019). The impact of timing of maternal influenza immunization on infant antibody levels at birth. Clin. Exp. Immunol. *195*, 139–152.

63. Marini, F., and Binder, H. (2019). pcaExplorer: an R/Bioconductor package for interacting with RNA-seq principal components. BMC Bioinf. *20*, 331.

64. Weiner, J., 3rd, and Domaszewska, T. (2016). tmod: an R package for general and multivariate enrichment analysis. PeerJ Preprints *4*, e2420v2421.

OMTN, Volume *34*

Supplemental information

Reducing cell intrinsic immunity to mRNA vaccine alters adaptive immune responses in mice

Ziyin Wang, Egon J. Jacobus, David C. Stirling, Stefanie Krumm, Katie E. Flight, Robert F. Cunliffe, Jonathan Mottl, Charanjit Singh, Lucy G. Mosscrop, Leticia Aragão Santiago, Annette B. Vogel, Katalin Kariko, Ugur Sahin, Stephanie Erbar, and John S. Tregoning

Supplemental Materials

Table S1: Immunisation with D1-uRNA induces a range of genes associated with inflammation. BALB/c mice were immunised intramuscularly with 10 µg mRNA encoding HA from H1 influenza. The mRNA was either D1-uRNA, cC1-modRNA or D1-modRNA; responses were compared to buffer only. Gene list of significant DEG in lymph nodes 24 hours after immunisation.

Figure S1

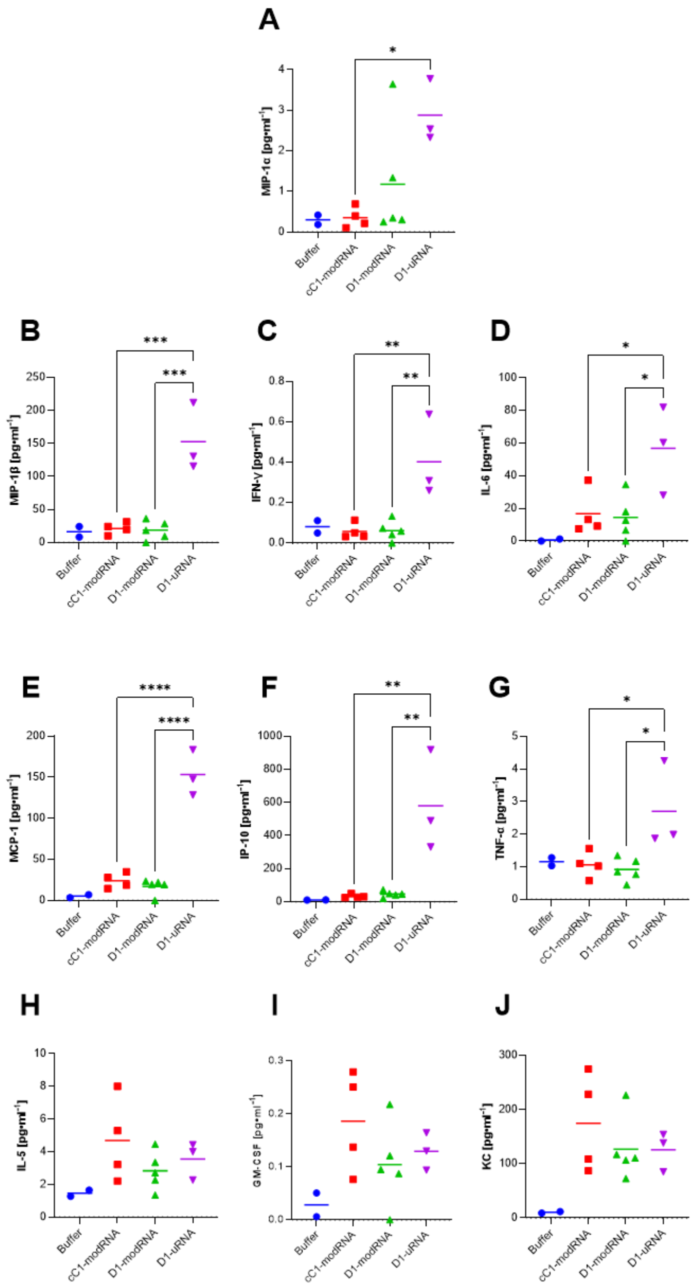

Figure S1. Immunisation with unmodified mRNA induces a significantly greater systemic inflammatory response 6 hours after immunisation. BALB/c mice were immunised intramuscularly with 10µg mRNA expressing HA from H1 influenza. The mRNA was either D1-uRNA, D1-modRNA, cC1-modRNA; responses were compared to buffer only. Blood was collected 6 hours after immunisation and measured for cytokines by MSD. Individual cytokines (A-J). * p<0.05, **p<0.01, *** p<0.001, **** p<0.0001 as indicated by One Way ANOVA with post test.

Figure S2

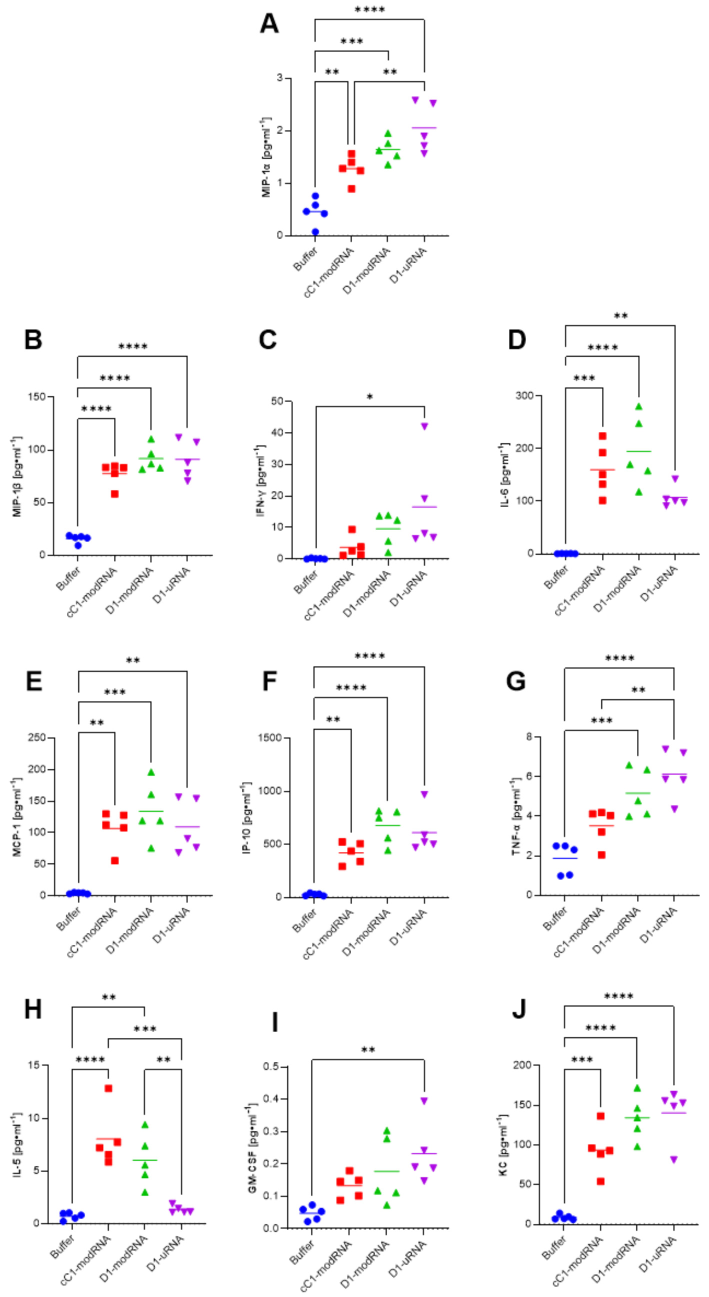

Figure S2. Immunisation with unmodified mRNA induces a significantly greater systemic inflammatory response 24 hours after immunisation. BALB/c mice were immunised intramuscularly with 10µg mRNA expressing HA from H1 influenza. The mRNA was either D1-uRNA, D1-modRNA, cC1-modRNA; responses were compared to buffer only. Blood was collected 24 hours after immunisation and measured for cytokines by MSD. Individual cytokines (A-J). * $p<0.05$, ** $p<0.01$, *** $p<0.001$, **** $p<0.0001$ as indicated by One Way ANOVA with post test.

Figure S3

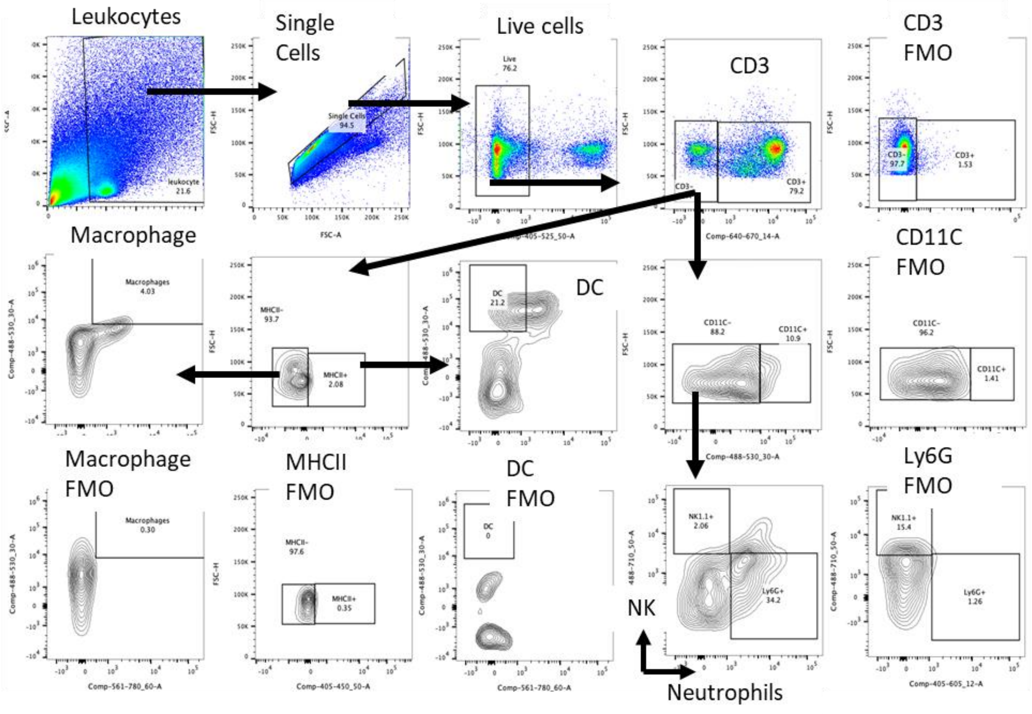

Figure S3. Gating strategy for flow cytometry analysis of leukocyte phenotypes in murine muscle and lymph nodes. Cells were isolated from lymph node and muscles after immunization and stained for flow cytometry. Analysis on FlowJo with sample gates demonstrated.
